# Parkinson’s disease recovery by GM1 oligosaccharide treatment in the *B4galnt1*^+/−^ mouse model

**DOI:** 10.1038/s41598-019-55885-2

**Published:** 2019-12-18

**Authors:** Elena Chiricozzi, Laura Mauri, Giulia Lunghi, Erika Di Biase, Maria Fazzari, Margherita Maggioni, Manuela Valsecchi, Simona Prioni, Nicoletta Loberto, Diego Yuri Pomè, Maria Grazia Ciampa, Pamela Fato, Gianluca Verlengia, Stefano Cattaneo, Robert Assini, Gusheng Wu, Samar Alselehdar, Robert W. Ledeen, Sandro Sonnino

**Affiliations:** 1 Department of Medical Biotechnology and Transcriptional Medicine, University of Milano, Milano, Italy; 2 School of Medicine, University Vita-Salute San Raffaele, Milano, Italy; 3 Department of Medical Sciences and National Institute of Neuroscience, University of Ferrara, Ferrara, Italy; 4 Rutgers, The State University of New Jersey, Newark, New Jersey USA; 5 Division of Neurochemistry, Department of Pharmacology, Physiology & Neuroscience, Rutgers New Jersey Medical School, Newark, NJ USA

**Keywords:** Diseases of the nervous system, Preclinical research

## Abstract

Given the recent *in vitro* discovery that the free soluble oligosaccharide of GM1 is the bioactive portion of GM1 for neurotrophic functions, we investigated its therapeutic potential in the *B4galnt1*^+/−^ mice, a model of sporadic Parkinson’s disease. We found that the GM1 oligosaccharide, systemically administered, reaches the brain and completely rescues the physical symptoms, reduces the abnormal nigral α-synuclein content, restores nigral tyrosine hydroxylase expression and striatal neurotransmitter levels, overlapping the wild-type condition. Thus, this study supports the idea that the Parkinson’s phenotype expressed by the *B4galnt1*^+/−^ mice is due to a reduced level of neuronal ganglioside content and lack of interactions between the oligosaccharide portion of GM1 with specific membrane proteins. It also points to the therapeutic potential of the GM1 oligosaccharide for treatment of sporadic Parkinson’s disease.

## Introduction

Gangliosides are key components of the neuronal plasma membranes^1–6^, where they participate to determine the membrane organization and to modulate protein functions, in many cases by interacting with their oligosaccharide moiety. Over the course of physiological aging, the ganglioside metabolic pathways (especially that of GM1) undergo progressive decline due to reduction of membrane ganglioside content and composition^7,8^, which can lead to progressive neurological dysfunction.

Accumulating evidence is pointing to a central role for subnormal levels of gangliosides as putative initiators of sporadic Parkinson’s disease (sPD) pathogenesis^9–12^. sPD patients present a reduction of GM1 ganglioside in the *substantia nigra*^9^, in the occipital cortex^13^ and various peripheral tissues, suggesting a GM1 systemic deficiency that would correlate with the systemic symptoms of sPD^12^. In neuromelanin-containing neurons in the sPD *substantia nigra* the reduced ganglioside content is accompanied by a significant deficient expression in two genes involved in ganglioside synthesis: B3GALT4 and ST3GAL2^10^. Several neuronal functions can become gradually compromised as the membrane GM1 content diminishes below a certain threshold with aging^14^ and/or under epigenetic influences. In particular, it has been hypothesized that the reduced level of plasma membrane GM1 in PD neurons can trigger the neurodegenerative process by a failure in neurotrophic signaling (i.e. GDNF)^13^ together with a reduction of GM1 interaction with α-synuclein (α-syn) that prevents aggregation of the latter^12,15,16^. Accordingly, as the population continues to age with a progressive decline of a-series gangliosides (GM1 and GD1a, its metabolic precursor, *via* plasma membrane bound sialidase Neu3), it can be expected that the number and percentage of persons developing sPD will multiply.

GM1 replacement therapy has shown modest but significant success in a monocentric controlled, delayed start trial in treated sPD patients^17^, acting as symptomatic and potentially disease modifier, since a partial restoration of dopamine (DA) transporter functional level in the striatum of GM1-treated subjects was reported^18^. Despite these suggestive positive evidences, the use of GM1 in clinical trials is severely hampered due to its low capacity to reach brain neurons. Gangliosides are amphiphilic compounds and in water solutions form micellar aggregates displaying very low aggregation concentration. The critical micellar concentration of GM1 is about 10^−9^ M^19^. Thus independently by the ganglioside concentration, the monomer concentration cannot be over 10^−9^ M. Only monomers are capable to insert into the cell membranes^20,21^ using their lipid moiety, the ceramide. Accordingly, a very minor quantity of injected GM1 overcomes the blood brain barrier and reaches the neurons. Thus to obtain a therapeutic effect, GM1 is injected in great amount increasing the possibility to inject significant amounts of contaminants^22,23^. The risk of GM1 protein contamination, due to its animal origin, and the completely disproved, but still discussed, relationship with Guillain-Barre syndrome^24–27^ inhibit serious consideration of GM1 therapeutic use.

The consequences of partial removal of GM1 and the more complex gangliosides, obtained from the heterozygous disruption of the *B4galnt1* gene (GM2/GD2 synthase), was a condition sufficient for these mice to develop PD phenotype: α-syn elevation and aggregation within central (CNS) and peripheral nervous (PNS) lesions, striatal degeneration and growing motor dysfunction^6,9,12,28,29^. Interestingly, *B4galnt1*^+/−^ mice subjected to GM1 replacement therapy by administration of semi-synthetic and brain-permeable GM1 derivative coded LIGA20 (with a dichloroacetyl group linked to the sphingosine amino group instead of the acyl chain) showed reversal of the pathological phenotype^9^. This result suggests that specific plasma membrane oligosaccharide–protein triggered pathways are required for the correct neuronal homeostasis.

Many studies on GM1 neurotrophic properties have been carried out using *in vitro* culture of neuroblastoma and pheochromocytoma cell lines, which differentiate into neuron like cells following GM1 exogenous administration^30–33^. The differentiative properties of GM1 have been associated to its monomeric insertion into the plasma membrane and to its interaction/modulation with membrane protein receptors, such as TrkA and RET, membrane ion channels and integrins^11,12,34^.

We recently reported that the soluble GM1 oligosaccharide administered to neuroblastoma cells replicates the neurotrophic and neuroprotective properties of the GM1 ganglioside^35–37^. The GM1 oligosaccharide added to the cell culture medium activates the TrkA auto-phosphorylation followed by the downstream MAPK signaling^35–37^. Molecular modelling suggested the formation of a very stable trimeric complex between GM1 oligosaccharide, TrkA and NGF^35^.

In this paper, we describe the results obtained by administering the soluble oligosaccharide of ganglioside GM1 to the heterozygous *B4galnt1*^+/−^ mouse, animal model of PD. During 4 weeks of oligosaccharide systemic administration, animals showed reversal of motor impairment, reduction of the aberrant levels of α-syn in the *substantia nigra* pars compacta (SNpc), recovery of nigral tyrosine hydroxylase (TH) expression and striatal DA level. These results are in favor of the development of a new human therapy of PD based on the administration of the GM1 soluble oligosaccharide.

## Results

### Identification of the [^3^H]OligoGM1 in the brain of treated WT mice

**Figure 1:**
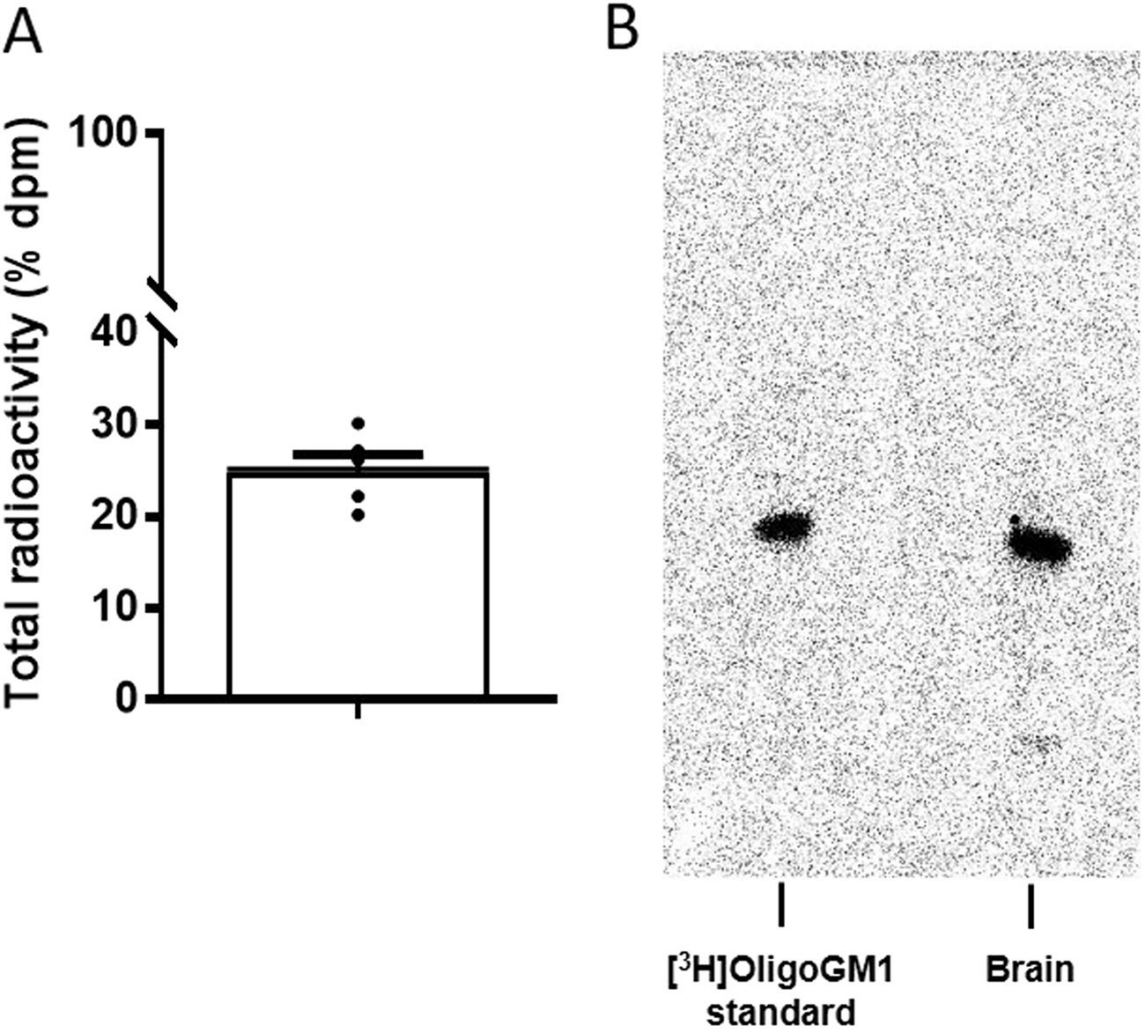
OligoGM1 penetrates into the brain. (**A**) Radioactivity associated to the brain mouse after injection of 1.3 × 10^7^ dpm [^3^H]OligoGM1. Data are expressed as mean ± SEM of five independent experiments (*n* = 5). (**B**) Representative image of the HPTLC separation of the radioactive material contained in the brain (*n* = 5). Lane 1: standard [^3^H]OligoGM1; Lane 2: soluble extract from brain homogenate derived from [^3^H]OligoGM1 injected mouse (24 h). Result is representative of those obtained for all the animals analyzed. HPTLC plates were developed with chloroform/methanol/0.2% CaCl_2_, 30:50:13 by vol. Tritium was detected with Beta-Imager 2000 instrument (Biospace) using an acquisition time of 16 h.

To understand if the OligoGM1 could reach the CNS, we administered [^3^H]OligoGM1 to wild-type (WT) mice. Mice were intraperitoneally (IP) injected with [^3^H]OligoGM1 (20 mg/kg plus 13 × 10^6^ dmp) and, 24 h following injection, brains were submitted to water soluble compounds and analyzed for the radioactivity and tritium labeled oligosaccharide contents. As shown in Fig. 1A, about 20% (±3.25 × 10^6^ dpm) of the total injected radioactivity (1.3 × 10^7^ dpm) was found associated to the brain. As reported (Fig. S1 of Supplementary) the large amount of radioactivity associated to the brain was non-volatile radioactivity, meaning that it is not associated to tritiated water generating upon the saccharide catabolism but rather it is associated to [^3^H]OligoGM1.

To further verify that the radioactivity was indeed corresponding to intact OligoGM1, we verify its metabolic stability by high-performance thin-layer chromatography (HPTLC) autoradiography using a BetaIMAGER ^T^RACER system (Biospace lab) and visualized using M3 vision software. As shown in Fig. 1B, metabolically stable [^3^H]OligoGM1 was found in the brain. No other radioactive compounds were identified considering that the detection limit of the instrument (BetaIMAGER ^T^RACER system, Biospace lab) is 15 spotted dpm.

### Restored motor activity in *B4galnt1*^+/−^ mice treated with OligoGM1

**Figure 2:**
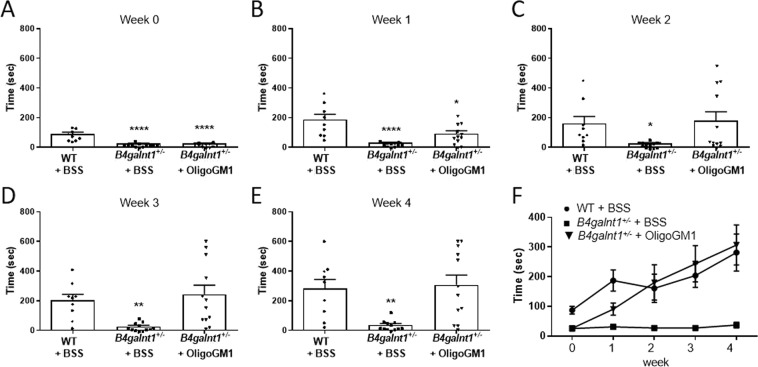
OligoGM1 effect on motor balance and coordination measured by grip duration test. (**A**–**E**) *B4galnt1*^+/−^ mice at 270 days of age were IP injected with 20 mg/kg OligoGM1 (*n* = 12) or with BSS (*n* = 12) daily for 4 weeks. WT mice (*n* = 9) of the same age were IP injected with BSS daily for 4 weeks. Data are expressed as mean ± SEM [one-way ANOVA followed by Bonferroni’s multiple comparisons test]. (**A**) Week 0, F_(2,30)_ = 31.36, ****p < 0.0001 *B4galnt1*^+/−^ + BSS and *B4galnt1*^+/−^ + OligoGM1 compared with WT. (**B**) Week 1, F_(2,30)_ = 13.30, ****p < 0.0001 *B4galnt1*^+/−^ + BSS compared with WT and *p < 0.05 B4galnt1^+/−^ + OligoGM1 compared with WT. (**C**) Week 2, F_(2,30)_ = 3.842, *p < 0.05 *B4galnt1*^+/−^ + BSS compared with WT and *B4galnt1*^+/−^ + OligoGM1. (**D**) Week 3, F_(2,30)_ = 7.546, **p < 0.01 *B4galnt1*^+/−^ + BSS compared with WT and *B4galnt1*^+/−^ + OligoGM1. (**E**) Week 4, F_(2,30)_ = 8.630, **p < 0.01 *B4galnt1*^+/−^ + BSS compared with WT and *B4galnt1*^+/−^ + OligoGM1; (**F**) overview of the 4 weeks’ treatment (●WT + BSS; ■ *B4galnt1*^+/−^ + BSS; ▼ *B4galnt1*^+/−^ + OligoGM1).

**Figure 3:**
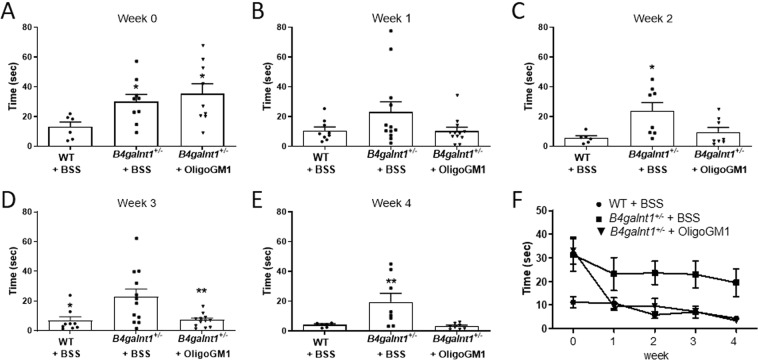
OligoGM1 effect on fine motor movement (sensorimotor) measured by irritant removal test. (**A**–**E**) *B4galnt1*^+/−^ mice at 270 days of age were IP injected with 20 mg/kg OligoGM1 (*n* = 12) or with BSS (*n* = 12) daily for 4 weeks. WT mice (*n* = 9) of the same age were IP injected with BSS daily for 4 weeks. Data are expressed as mean ± SEM [one-way ANOVA followed by Bonferroni’s multiple comparisons test]. (**A**) Week 0, F_(2,21)_ = 3.710, *p < 0.05 *B4galnt1*^+/−^ + BSS and *B4galnt1*^+/−^ + OligoGM1 compared with WT. (**B**) Week 1, no significant difference between *B4galnt1*^+/−^ + BSS or + OligoGM1 compared with WT. (**C**) Week 2, F_(2,19)_ = 5.256, *p < 0.05 *B4galnt1*^+/−^ + BSS compared with WT and *B4galnt1*^+/−^ + OligoGM1. (**D**) Week 3, F_(2,30)_ = 6.783, *p < 0.05 *B4galnt1*^+/−^ + BSS compared with WT and **p < 0.01 *B4galnt1*^+/−^ + BSS compared with *B4galnt1*^+/−^ + OligoGM1. (**E**) Week 4, F_(2,19)_ = 6.027, **p < 0.01 *B4galnt1*^+/−^ + BSS compared with WT and *B4galnt1*^+/−^ + OligoGM1; (**F**) overview of the 4 weeks’ treatment (●WT + BSS; ■ *B4galnt1*^+/−^ + BSS; ▼ *B4galnt1*^+/−^ + OligoGM1).

**Figure 4:**
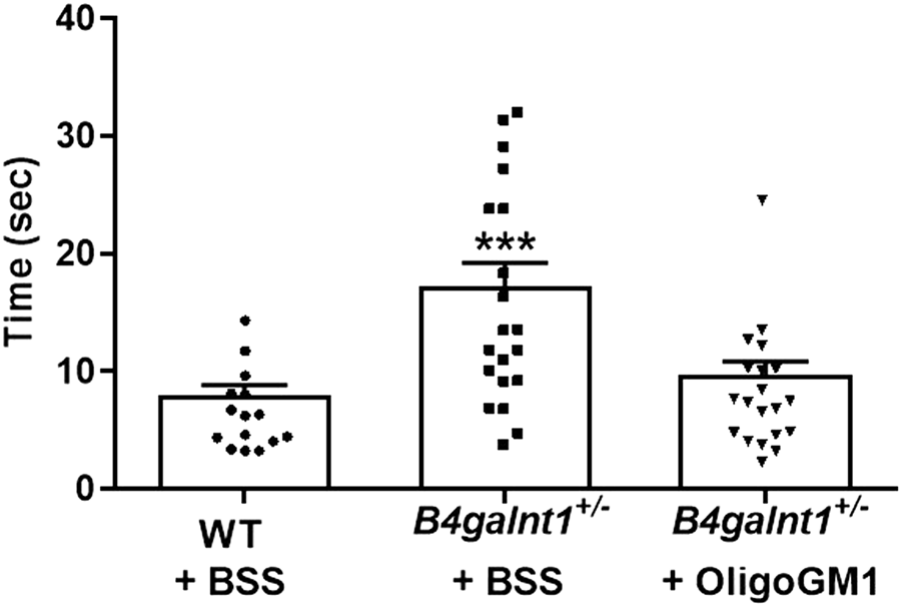
OligoGM1 effect on motor balance and coordination measured by pole climbing test. *B4galnt1*^+/−^ mice at 270 days of age were IP injected with 20 mg/kg OligoGM1 (*n* = 12) or with BSS (*n* = 12) daily for 4 weeks. WT mice (*n* = 9) of the same age were IP injected with BSS daily for 4 weeks. Data are expressed as mean ± SEM: F_(2,52)_ = 10.37, ***p < 0.001 *B4galnt1*^+/−^ + BSS compared with WT and *B4galnt1*^+/−^ + OligoGM1 [one-way ANOVA followed by Bonferroni’s multiple comparisons test].

OligoGM1 (20 mg/Kg) was daily IP injected to *B4galnt1*^+/−^ mice for 4 weeks. To evaluate its effect on motor coordination, balance and fine motor movement, grip duration and irritant removal tests were assessed weekly^9,13,28^. Before starting the treatments, the physical impairment of animals was tested to record the basal state for both “grip” and “irritant removal” tests, in comparison with WT animals (Figs. 2 and 3). The graphs depicted in Fig. 2 and in Fig. 3 clearly show a significant recovery in motor behavior of *B4galnt1*^+/−^ treated mice. Moreover, mice were tested with pole climbing for motor coordination and balance (Fig. 4) but only at the end of treatment to avoid that the learning ability could influence the success of the test. Figures 2–4 show that the treated *B4galnt1*^+/−^ mice rapidly recovered motor functions, some of them immediately after the first week of injections. OligoGM1 treated PD animals showed motor functions comparable with those of WT animals following 4 weeks of daily treatment. No significant difference was found between male and female mice in response to the treatment (Fig. S2 of Supplementary).

### Reduction in a-series gangliosides in *B4galnt1*^+/−^ mice

Following the 4 weeks of treatment, the ganglioside pattern and content in mice brain cortex and cerebellum were analyzed following brain lipid extraction, ganglioside purification, ganglioside separation by HPTLC and ganglioside revelation by colorimetric detection^39–41^. WT and *B4galnt1*^+/−^ mice treated with balanced saline solution (BSS), and *B4galnt1*^+/−^ treated with the OligoGM1 were analyzed.

**Figure 5:**
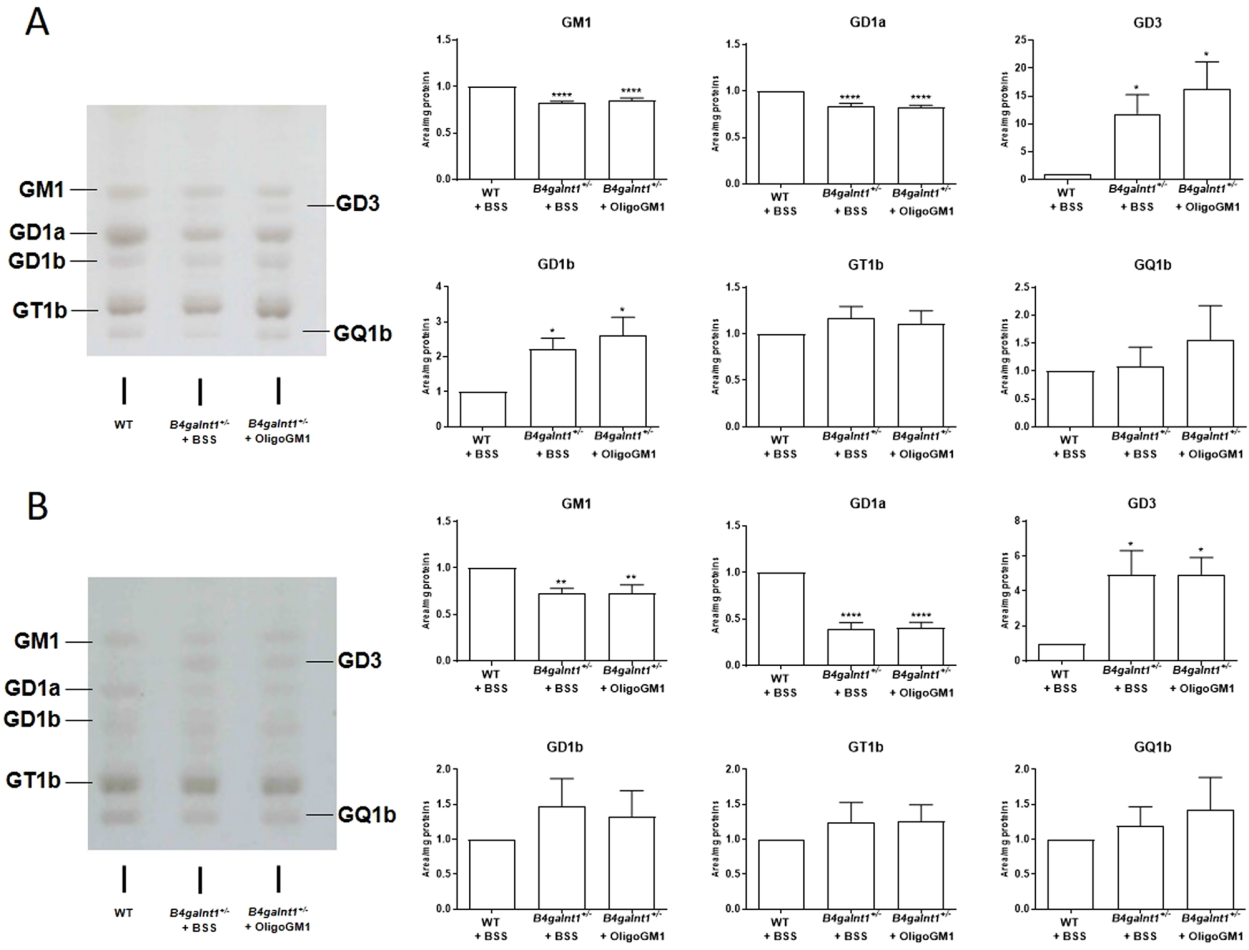
Ganglioside content in brain cortex (**A**) and cerebellum (**B**). Ganglioside patterns resolved by HPTLC in the presence of specific standards and revealed by Ehrlich spray reagent. On the left: HPTLC representative image. On the right: relative quantitation of the intensity of each ganglioside band normalized on the protein content. Patterns are representative of those obtained for all the animals analyzed (WT, *n* = 5; *B4galnt1*^+/−^ + BSS, *n* = 8 *B4galnt1*^+/−^ + OligoGM1, *n* = 8). Data are express as fold increase or decrease over WT of mean ± SEM: (**A)** GM1: F_(2,21)_ = 23.54 ****p < 0.0001, GD1a: F_(2,21)_ = 18.35 ****p < 0.0001, GD3: F_(2,21)_ = 5.178, GD1b F_(2,21)_ = 5.762 *p < 0.05 WT vs *B4galnt1*^+/−^ (one-way ANOVA, followed by Bonferroni’s post-hoc test); (**B**) GM1: F_(2,21)_ = 6.948 **p < 0.01, GD1a: F_(2,21)_ = 46.01 ****p < 0.0001, GD3: F_(2,18)_ = 5.479 *p < 0.05 WT vs *B4galnt1*^+/−^ (one-way ANOVA, followed by Bonferroni’s post-hoc test).

The *B4galnt1*^+/−^ mouse brains had reduced amount of GM1 and GD1a with respect to WTs in both brain regions examined (Fig. 5). In parallel, we found a fair accumulation of GD3 in cerebellum and cerebral cortex of *B4galnt1*^+/−^ mice with respect to WTs (Fig. 5). With the exception of GD1b, which was elevated in the cortex of *B4galnt1*^+/−^ mice, the content of b-series gangliosides was not affected, suggesting that the amount of GD3 ganglioside available results in an optimal substrate level for the galactosaminyl transferase. No differences in ganglioside pattern and content were found in the brain of *B4galnt1*^+/−^ animals treated for 4 weeks with saline solution or OligoGM1, indicating that the treatment is not capable to act on the metabolic pathway of gangliosides (Fig. S3 of Supplementary).

These results confirm the reduction of gangliosides GM1 and GD1a in the *B4galnt1*^+/−^ brain mice^9^ and show that the recovery of motor functions in *B4galnt1*^+/−^ animals following oligosaccharide treatment was not associated to recovery of gangliosides GM1 and GD1a expression, due to activation of residual galactosaminyl transferase enzyme.

### Reduction of the aberrant α-syn content in the SNpc of *B4galnt1*^+/−^ mice treated with OligoGM1

**Figure 6:**
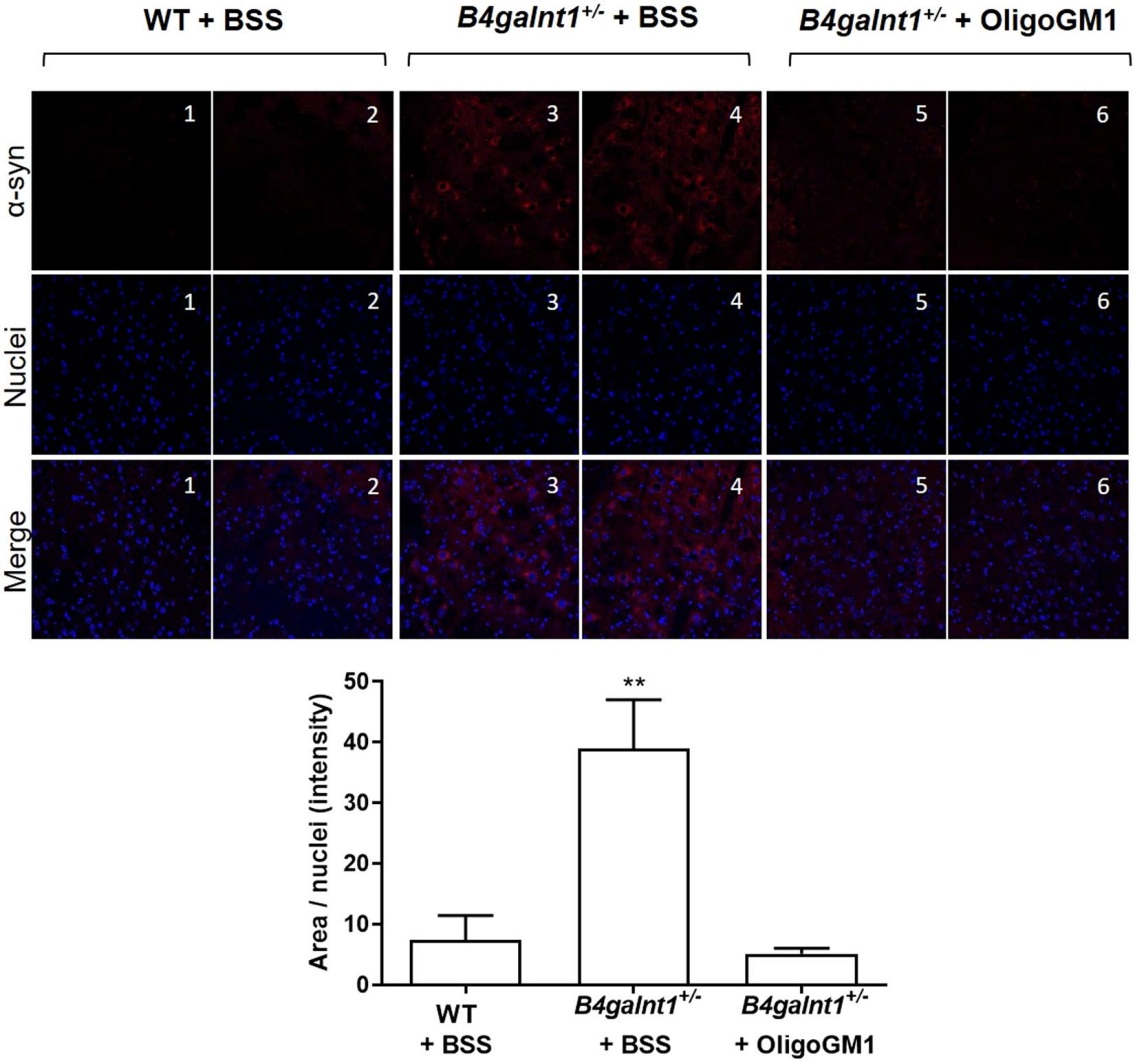
α-syn content within *substantia nigra*. On the top: representative fluorescent IHC images of α-syn (red) and nuclei (blue) immunolabeling in the SNpc. (**1–2)** WT + BSS (*n* = 4); (**3–4**) *B4galnt1*^+/−^ + BSS, (*n* = 4) **(5–6)** *B4galnt1*^+/−^ + OligoGM1, *n* = 4). On the bottom: quantification of α-syn normalized on nuclei area (*n* = 4 for group; 8–10 brain sections/animal). Data are expressed as mean ± SEM (F_(2,32)_ = 7.7, **p < 0.01 *B4galnt1*^+/−^ + BSS compared with WT and *B4galnt1*^+/−^ + OligoGM1; one-way ANOVA followed by Bonferroni’s multiple comparisons test).

A key feature of the *B4galnt1*^+/−^ mice was marked elevation of α-syn in the SNpc^9^. To understand if the restored motor activity was accompanied by biochemical rescue of α-syn physiological levels, we performed fluorescent immunohistochemistry (IHC) analysis of nigral α-syn. We confirmed the increased content of α-syn in the *B4galnt1*^+/−^ mice (Fig. 6), reproducing what was previously reported^9^. Interestingly, the OligoGM1 treated *B4galnt1*^+/−^ mice were found with significantly reduced levels of α-syn overall reaching the WT situation (Fig. 6). Although we did not quantify the level of Ser129-phosphorylated α-syn, we found by IHC the positivity of α-syn to the Ser129-phosphorylation in the untreated *B4galnt1*^+/−^ mice at the level of SNpc (Fig. S4, panel A of Supplementary). Importantly, the positive signal was less intense in the OligoGM1 treated *B4galnt1*^+/−^ and, as expected, in WT mice (Fig. S4, panel A of Supplementary). Finally, immunoblotting analysis showed levels of Ser129-phosphorylated α-syn in OligoGM1 treated animals similar to those of WT mice, and lower compared to saline-treated animals (Fig. S4, panel B of Supplementary).

### Recovery of the TH expression in the SNpc *substantia nigra* of *B4galnt1*^+/−^ mice treated with OligoGM1

**Figure 7:**
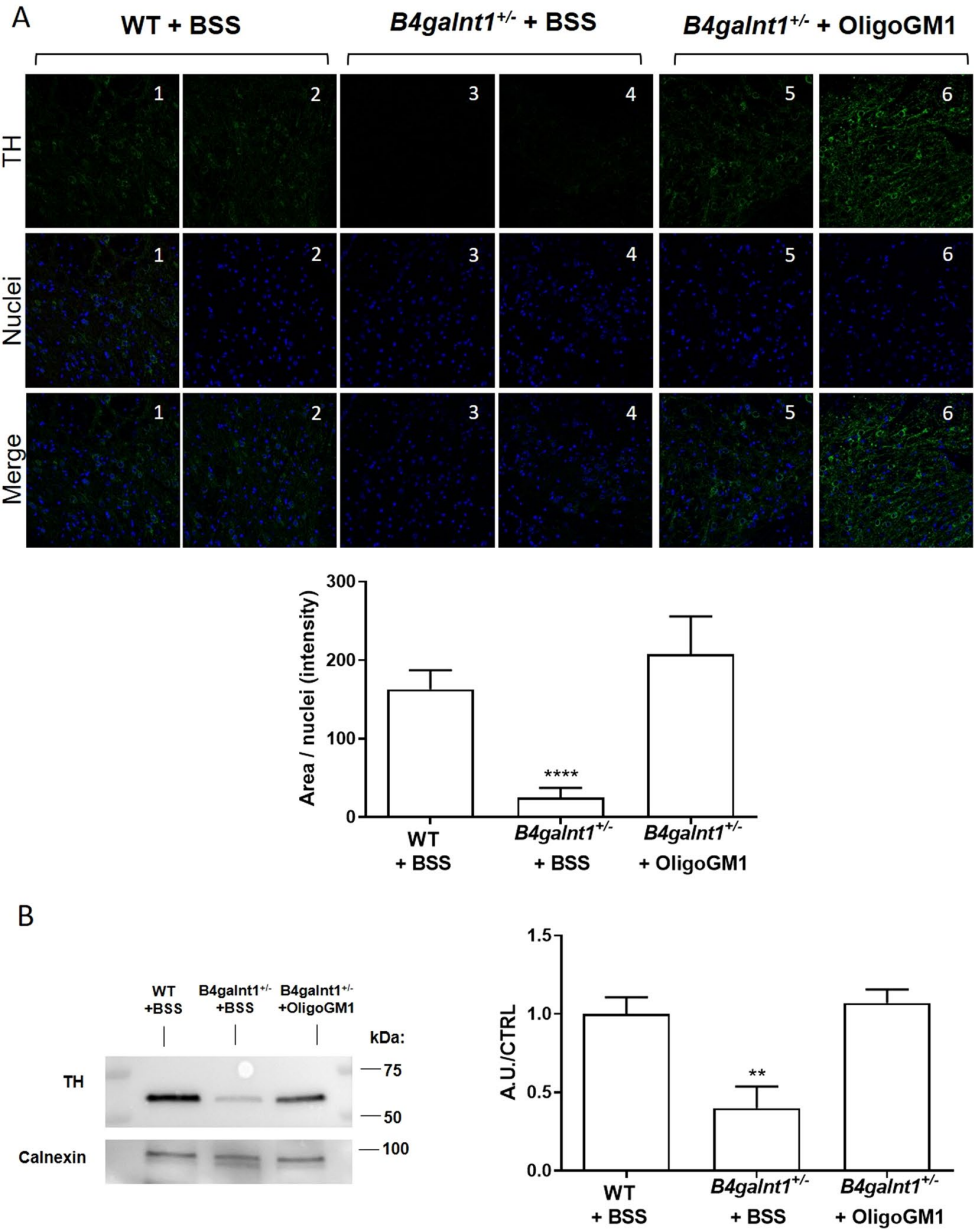
TH expression in the *substantia nigra*. (**A**) On the top: representative fluorescent IHC images of TH^+^ neurons (red) and nuclei (blue) immunolabeling in the SNpc. (**1–2)** WT + BSS (*n* = 4); (**3**–**4**) *B4galnt1*^+/−^ + BSS, (*n* = 4) (**5–6**) *B4galnt1*^+/−^ + OligoGM1, *n* = 4). On the bottom: quantification of the TH^+^ neurons normalized on nuclei area (*n* = 4 for group; 8–10 brain/sections animal). Data are expressed as mean ± SEM (F_(2,32)_ = 17.26, ****p < 0.001 *B4galnt1*^+/−^ + BSS compared with WT and *B4galnt1*^+/−^ + OligoGM1; one-way ANOVA followed by Bonferroni’s multiple comparisons test). (**B**) On the left: representative immunoblotting images of TH and calnexin are shown after cropping (full length images of blot are presented as Supplementary Fig. S8). On the right: Semi-quantitative analysis of nigral TH expression related to calnexin level (*n* = 4). Data are expressed as fold increase or decrease over the control (WT) of the mean ± SEM (F_(2,8)_ = 10.34, **p < 0.01 *B4galnt1*^+/−^ + BSS compared with WT and *B4galnt1*^+/−^ + OligoGM1; one-way ANOVA followed by Bonferroni’s multiple comparisons test).

It has been reported that the *B4galnt1* heterozygous disruption causes the degeneration of TH expressing neurons within the SNpc as well as the reduction of TH expression level^9^. To verify if OligoGM1 could restore the TH expression, we performed fluorescent IHC analysis on SNpc. As previously reported^9^, we found a decrease level of TH expression in *B4galnt1*^+/−^ mice, which was rescued by OligoGM1 treatment, with restoration to WT levels (Fig. 7A). Additionally, immunoblotting evaluation confirmed a significant recovery of nigral TH expression in *B4galnt1*^+/−^ mice treated with OligoGM1 compared to the WT level (Fig. 7B).

### Restoration of striatal neurotransmitter levels in *B4galnt1*^+/−^ mice treated with OligoGM1

**Figure 8:**
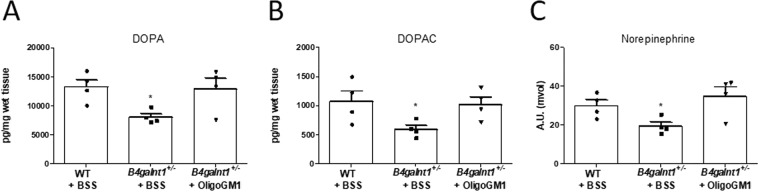
Striatal neurotransmitter content. (**A**,**B**) Striatal tissue DA and DOPAC content are expressed as pico-grams per mg of wet weight of tissue. Data are expressed as mean ± SEM: DA, F_(2,9)_ = 4.61, *p < 0.05 *B4galnt1*^+/−^ + BSS compared with WT and *B4galnt1*^+/−^ + OligoGM1; DOPAC, F_(2,9)_ = 4.834, *p < 0.05 *B4galnt1*^+/−^ + BSS compared with WT and *B4galnt1*^+/−^ + OligoGM1 (*n* = 4, one-way ANOVA followed by Bonferroni’s multiple comparisons test). (**C**) Striatal tissue norepinephrine content data are expressed as arbitrary units (mvolt). Data are expressed as mean ± SEM (*n* = 4, F_(2,9)_ = 4.915, *p < 0.05 *B4galnt1*^+/−^ + BSS compared with WT and *B4galnt1*^+/−^ + OligoGM1; one-way ANOVA followed by Bonferroni’s multiple comparisons test).

The homozygous *B4galnt1*^−/−^ mice were previously found to have decreased levels of DA as well as its metabolite, 3,4-dihydroxyphenylacetic acid (DOPAC), in the striatum^28^. Thus, in order to further validate our findings and to determine whether the OligoGM1 could rescue the pathological phenotype by promoting the synthesis of striatal neurotransmitters, we performed neurochemical analysis with electrochemical detection high performance liquid chromatography (ED-HPLC). We confirmed that striatal levels of DA, DOPAC and norepinephrine are diminished in BSS treated *B4galnt1*^+/−^ mice with respect to WT mice (Fig. 8, Fig. S5 of Supplementary). Importantly, we found that the levels of DA, DOPAC and of norepinephrine were rescued in OligoGM1 treated *B4galnt1*^+/−^ mice (Fig. 8, Fig. S5 of Supplementary).

## Discussion

Unlike familial forms, sPD has no cogent theory that summarizes the disparate data into a compelling narrative regarding its etiology^42^. Although various hypotheses have been suggested, none has successfully reconciled the collected data that explain the diverse central and peripheral manifestations of PD. α-Syn is generally considered to have a central role in PD, but as yet there is no explanation as to what causes its elevation and resultant aggregation in sporadic PD coinciding with progressive impairment of the neuronal dopaminergic system^12^.

Recently, a theory has emerged that defines a central role for ganglioside GM1 which levels diminish with aging^7,8^ and/or under epigenetic influences; it was proposed that in some individuals, presumably those starting life with lower levels, GM1 falls below a threshold level necessary to maintain TH neuron viability thereby gradually leading to sPD^12^. This would be the consequence of the numerous essential neuronal functions of GM1^14,23^. Considering the cases reported so far, sPD patients show GM1 deficiency in the *substantia nigra*^9^, the occipital cortex^13^, and in various peripheral tissues^12^, suggesting a systemic GM1 deficiency. Moreover, a significantly deficient expression in genes involved in ganglioside synthesis (B3GALT4, ST3GAL2) was reported in neuromelanin-containing neurons (but not in other neuronal types) in the *substantia nigra* of sPD subjects^10^. These findings suggest a ganglioside systemic deficiency that would correlate with the systemic symptoms of sPD.

It has been hypothesized that subnormal levels of a-series ganglioside (GM1/GD1a) can gradually compromise several neuronal functions^14,23^. In particular, the reduced level of plasma membrane GM1 in sPD neurons can trigger the neurodegenerative process by a failure in trophic signaling via GDNF^13^ together with a reduction of clearance promoting the α-syn accumulation^6,14,43^. Most relevant in regard to neurodegeneration are neurotrophic factors whose receptors require GM1 association to facilitate lifelong survival of neurons. An example of this was the finding that GM1 binds with high-affinity to TrkA, the NGF receptor, and promotes tyrosine autophosphorylation: the starting event leading to neurite elongation^34,44–47^. Moreover, a similar GM1 partnership has been reported for the GDNF receptor, comprising RET receptor and GPI-anchored co-receptor GFRα^12,13^. Deficiency of RET phosphorylation, an indicator of failed GDNF signaling, was identified in the *substantia nigra* of sPD subjects and also in the GM1-deficient *B4Galnt1*^+/−^ mice; in the latter GDNF signaling was restored by LIGA20, a membrane-permeable analog of GM1^13^. In addition, GM1 displays binding to the soluble α-syn, and the interaction between GM1 and α-syn has been proposed to prevent α-syn aggregation and deposition^15,16^. Structural studies have shown the existence of α-syn region defined as the ganglioside binding region^15^. *In vitro* experiments revealed that N-alpha-acetylation of α-syn strengthened the interaction with GM1 and its resistance to aggregation^16^. In addition to this, free α-syn inhibits Nurr1, necessary for the expression and functioning of GDNF signaling^48^. Significantly, application of GM1 to a rat α-syn model of PD reduced α-syn aggregation^43^.Thus, the reduction of brain GM1 is claimed to be responsible for the onset of sPD.

GM1 replacement therapy has shown modest but significant success in a monocentric controlled, delayed start trial in treated sPD patients, acting as symptomatic and potentially disease modifier^17^; significantly, partial restoration of DA transporter functional level in the striatum of GM1-treated subjects was reported^18^. These results strongly support the idea that the loss of GM1-sustained trophism is the crucial point in sPD pathogenesis, so much so that restoring its brain level ameliorates symptoms and slows the disease progression. However, the great amount of GM1 necessary to obtain results, its animal origin and its discussed immunogenicity^22,23^ have questioned GM1 as a suitable drug for human therapy.

Beneficial results were gained in *B4galnt1* GM1-deficient mice treated with a semi-synthetic, brain-permeable GM1 ganglioside analog, LIGA20^9,12,13^. This GM1 analogue was prepared by FIDIA-Farmaceutici S.p.a. (Italy) as membrane permeable ganglioside analog capable to traverse the blood brain barrier. In those mice, LIGA20 treatment could also reduce accumulation of α-syn in *substantia nigra*^9,12^. Most important in this study is the fact that the use of a more hydrophilic GM1 derivative, modified on the ceramide moiety with a dichloroacetyl group instead of the C_18_ acyl chain, but keeping the entire oligosaccharide chain intact, still maintains trophic potential, supporting the hypothesis that only specific plasma membrane oligosaccharide–protein triggered pathway is required for the correct neuronal homeostasis^9,12^ and not the lipid moiety. Human therapy based on that particular compound cannot be approached due to the toxicity of the dichloroacetyl moiety.

It was notable that plasma membrane GM1 increase by intraventricular injection of Vibrio cholerae sialidase exerts a neuroprotective effect on the damaged nigrostriatal DA system of MPTP mice^49^. This enzyme removes sialic acid residues from polysialogangliosides (e.g. GD1a) in the brain, thus increasing plasma membrane GM1. In this scenario, the idea emerges that plasma membrane GM1 oligosaccharide decline could be the trigger for sustained pathways impairment as the etiopathogenetic target event of PD.

Recently, we showed in neuronal cells that, within the entire GM1 molecule, the oligosaccharide chain is the actual moiety responsible for GM1 neurotrophic properties^35–37^. GM1 oligosaccharide directly interacts with the NGF receptor TrkA, leading to ERK1/2 downstream pathway activation, to cell differentiation and to sustained neuroprotection from MPTP toxicity. Proteomic analysis revealed a broad spectrum of molecular events prompted by the OligoGM1 involving calcium regulation, antioxidant mechanism, mitochondrial bioenergetics, and anti-inflammatory response^36^.

Considering all our *in vitro* evidence about the OligoGM1 neurotrophic potential, we wondered if the OligoGM1 could be used to replace GM1 functions using the *B4galnt1*^+/−^ mouse as *in vivo* approach. This model, obtained by the heterozygous disruption of the B4galnt1 gene (GM2/GD2 synthase) causing partial deletion of GM1, represents an accurate recapitulation of the human sPD condition. These mice, with reduced brain content of GM1 and GD1a (especially in the SNpc^9^) with respect to the WT condition, develop progressively the sPD phenotype: alteration of neurotrophic signaling, SNpc α-syn elevation and aggregation within CNS and PNS lesions, loss of TH-expressing neurons in the SNpc, striatal degeneration, and increasing motor dysfunction^9,12,13,28^.

Firstly, we verified the capacity of the OligoGM1 to cross the blood brain barrier and stably reach the CNS, postponing a detailed study on its pharmacological distribution to the future. This study demonstrated recovery of normal motor function in the *B4galnt1*^+/−^ mice by OligoGM1 treatment, as shown with three movement tests. Similarly, neuropathological lesions were largely resolved by OligoGM1 in relation to nigral TH-expressing neurons and neurotransmitter (DA, norepinephrine) levels in the striatum. OligoGM1-treated animals also showed a significant reduction of the abnormal α-syn content compared to untreated animals at the level of SNpc. Although we performed a limited examination regarding the toxic phosphorylated form of α-syn, which specifically represents the dominant pathological modification of α-syn in familial and sporadic Lewy body diseases, as well as in transgenic animal models of synucleinopathies^50–52^, we found a reduction expression in the α-syn phosphorylation (Ser129) in OligoGM1-treated *B4galnt1*^+/−^ mice compared to untreated mice. Now, follow-up studies are necessary to study in detail the relationship between OligoGM1 administration and α-syn phosphorylation and aggregation.

Importantly, we did not find any difference in ganglioside content between OligoGM1 and BSS treated *B4galnt1*^+/−^ mice, suggesting that the OligoGM1 is not able to influence the ganglioside metabolic pathway.

Although our study does not explain the exact mechanism by which the OligoGM1 rescues the PD phenotype, it strongly supports the idea that it could account for the GM1 neurotrophic function. Our hypothesis is that the sPD pathogenesis is fundamentally due to plasma membrane GM1 deficiency that causes the alteration/loss of molecular interaction between the oligosaccharide portion of GM1 and specific proteins, giving rise to the gradual impairment of several functions. We suggest that in sPD neurons, the reduced level of GM1, and hence of its oligosaccharide, could trigger the neurodegenerative process by a failure in trophic signals together with a reduction of α-syn clearance.

The idea that the age-dependent GM1 deficiency could be the trigger for idiopathic PD is attractive, not only because it is supported by preclinical data, but also because it recognizes that GM1 functions as a key node in a network of regulated processes and its deficiency would affect the neuronal homeostasis in multi-modal ways^12,14^. The GM1 decrease could drive trophic signaling failure and α-syn accumulation, leading in turn to glia/microglia activation, oxidative stress, mitochondria dysfunction and excitotoxicity, resulting in the onset of cell death. Our recent data^36^ showed that GM1 oligosaccharide activates a broad range of neuroprotective biochemical pathways in neuroblastoma cells and prevents their death caused by toxic molecules. The latter findings, together with the results of the present work, concur in setting a reasonable basis for considering GM1 oligosaccharide as an agent that overcomes GM1 pharmacological limits and could display significant therapeutic benefit for sPD.

## Materials and Methods

### Materials

Commercial chemicals were of the highest purity available, common solvents were distilled before use and water was doubly distilled in a glass apparatus.

Paraformaldehyde (PFA), BSS, sucrose, fetal calf serum, Triton X-100, bovine serum albumin, trypan blue, perchloric acid, sodium acetate, sodium octyl sulfate, EDTA, DA, DOPAC and DAPI were from Sigma-Aldrich (St. Louis, MO, USA). Phosphate buffered saline (PBS) was from EuroClone. 4–20% Mini-PROTEAN TGX Precast Protein Gels, Turbo Polyvinylidene difluoride (PVDF) Mini-Midi membrane and DC protein assay kit were from Bio-Rad (Hercules, CA, USA). HPTLC plates were from Merck (Frankfurt, Germany). Optima cutting temperature (OCT) was from VWR (Radnor, PA, USA). Dako fluorescent mounting medium was from Dako (Glostrup, Denmark). Chemiluminescent kit for western blot was from Cyanagen (Bologna, Italy). Ultima gold was from PerkinElmer (Waltham, MA, USA).

### GM1 ganglioside and GM1 oligosaccharide preparation

GM1 ganglioside was purified from the total ganglioside mixture extracted from fresh pig brains collected at the slaughterhouse of the Galbani company (Melzo, Italy), according to the procedure developed previously^53^. The ganglioside mixture, 5 g as sialic acid, was dissolved in prewarmed (36 °C) 500 mL of 0.05 M sodium acetate, 1 mM CaCl_2_ buffer, pH 5.5. Vibrio cholerae sialidase (1 unit) was added to the solution every 12 h^54^. Incubation at 36 °C and under magnetic stirring was maintained for two days, and the solution dialyzed at 23 °C for 4 days against 10 L of water changed 5 times a day. The sialidase treated ganglioside mixture was subjected to 150 cm × 2 cm silica gel 100 column chromatography equilibrated and eluted with chloroform-methanol-water, 60:35:5 by vol. The fractions containing GM1, identified by TLC, were pooled, dried and submitted to a further column chromatographic purification using the above experimental conditions. Fractions containing pure GM1 were collected and dried. The residue was dissolved in chloroform-methanol (2:1 v/v) and precipitated by adding 4 volumes of cold acetone. After centrifugation (15,000 × g) the GM1 pellet was separated from the acetone, dried, dissolved in 50 mL of deionized water and lyophilized giving 1350 mg of white powder which was stored at −20 °C.

GM1 containing tritium at position 6 of external galactose was prepared by enzymatic oxidation with galactose oxidase followed by reduction with sodium boro[^3^H]hydride^55^.

The oligosaccharides II^3^Neu5Ac-Gg_4_ and II^3^Neu5Ac-[6-^3^H(*IVGal*)]Gg_4_ (OligoGM1 and [^3^H]OligoGM1, respectively) were prepared by ozonolysis followed by alkaline degradation^56^ of GM1 and [^3^H]GM1, respectively. Minor changes for the alkaline degradation were introduced. Briefly, GM1 ganglioside or [^3^H]GM1 gangliosides was dissolved in the minimum required methanol and slowly saturated with and maintained under ozone at 23 °C for 6 h under continuous stirring. The solvent was then evaporated under vacuum and the residue brought immediately to pH 10.5–11.0 by addition of triethylamine. After solvent evaporation, GM1 oligosaccharide or [^3^H]GM1 oligosaccharide was purified by flash chromatography using chloroform/methanol/2-propanol/water 60:35:5:5 v/v/v/v as eluent. GM1 oligosaccharide was dissolved in methanol and stored at 4 °C.

NMR, Mass Spectrometry and HPTLC analyses showed a purity over 99% for the prepared OligoGM1 (Fig. S6 of Supplementary Files). HPTLC followed by radio-imaging showed a purity over 98% for the [^3^H]OligoGM1 (Fig. 1B).

### Animal

All animal procedures were approved by the Rutgers University and conducted in accordance with Institutional Animal Care and Use Committee guidelines. Mice were handled following the Ethical Guidelines for Treatment of Laboratory Animals of Rutgers University.

Animals were housed with a 12 h light/12 h dark cycle in groups of five in plastic cages with *ad libitum* access to food and water.

A breeding pair of heterozygotes with disrupted gene for *B4galnt1* (C57BL/6 background), created by Dr. Richard Proia and coworkers^57^, was provided as gift by Dr. Ronald Schnaar (Johns Hopkins University, Baltimore, MD, USA). Generation of *B4galnt1*^+/−^ − C57BL/6 J heterozygous mice has been described previously^9^. All mice were backcrossed for 10 generations in a C57BL/6J background. For all experiments, WT mice with the same genetic background bred and housed in the same animal facility were used as controls. All mice were 8–9 months old at the start of the experiments. Animals were genotyped as previously reported^58^. Both male (35 to 45 g) and female (25 to 35 g) mice have been included in the study to maintain the variability introduced by sex factors, reported for PD^59^.

### Animals treatment

OligoGM1 was dissolved in BSS at a concentration of 4 mg/mL and was injected intraperitoneal (IP) at 20 mg/kg body weight, daily for 4 weeks.

Since it is already known that systemically administered GM1 fails to cross the blood brain barrier^60^, in our experiments we chose an arbitrary and lower dose of the oligosaccharide molecule with respect to the quantity of the lipid (GM1) used in peripheral neurodegenerative experiments in the past (30 mg/kg)^61^.

*B4galnt1*^+/−^ mice were randomly assigned to receive daily BSS (*n* = 12, female *n* = 6, male *n* = 6) or OligoGM1 (*n* = 12, female *n* = 6, male *n* = 6). No animal was excluded from analysis. At the conclusion of the study (treatment and parallel behavioral testing), animals were euthanized, and randomly divided for the biochemical analysis.

For IHC analysis, four brain for each group [WT + BSS *n* = 4 (2 male and 2 female); *B4galnt1*^+/−^ + BSS *n* = 4 (2 male and 2 female); *B4galnt1*^+/−^ + OligoGM1, *n* = 4 (2 male and 2 female)] were fixed in 4% PFA. For neurochemical, ganglioside and western blotting studies the same animals were used to collect different brain area. Specifically:

- for neurochemical analysis striatal samples (both sides) were rapidly removed and immediately frozen for later analysis from four brain for each group [WT + BSS *n* = 4 (2 male and 2 female); *B4galnt1*^+/−^ + BSS *n* = 4 (2 male and 2 female); *B4galnt1*^+/−^ + OligoGM1 *n* = 4 (2 male and 2 female)].
- for ganglioside analysis, cortex and cerebellum samples were rapidly collected and immediately frozen for later analysis [WT + BSS *n* = 5 (3 male and 2 female), B4galnt1^+/−^ + BSS *n* = 8 (4 male and 4 female), *B4galnt1*^+/−^ + OligoGM1, *n* = 8 (4 male and 4 female)];
- for western blotting *substantia nigra* samples were rapidly removed and immediately frozen for later analysis from four brain for each group [WT + BSS *n* = 4 (2 male and 2 female); *B4galnt1*^+/−^ + BSS *n* = 4 (2 male and 2 female); *B4galnt1*^+/−^ + OligoGM1 *n* = 4 (2 male and 2 female)].

### Behavioral experimental procedures

The sample size was predetermined using G*Power software and *a priori* analysis^9,28^. The effect size was determined for each experiment considering the difference we expected to see with respect to control group and the standard deviations that we derived from previous published data. Before starting behavioral experiments, each animal was handled for five days. Each experiment was performed during the light cycle and in a separate behavioral testing room after a 30-min-period of acclimation. During the test performing the rater was blind as to the experimental condition of the animal.

*Grip duration test* was used to test motor coordination and balance^38^. Briefly, each mouse was suspended by its forepaws on a horizontal rounded metal bar (diameter 3 mm) positioned 50 cm above a foam pillow and the mouse’s tail is gently held by experimenter to prevent climbing with hind legs^9,13,62^. The hang time to fall is measured up to 600 s for each trial in three consecutive trials, with a rest interval of 30 min. The hang times were then averaged for each mouse. Mice were tested for grip duration before starting the treatment to measure the basal state and once a week over the treatments.

*Irritant remova*l *test* was assessed to determine motor response to sensory stimulus^9,13,38,63^. Each mouse is restrained and a small piece of adhesive is applied on the snout using forceps. The mouse is placed in a cage and a timer is set to record how long it takes each mouse to remove the sticker, typically with their forepaws. The maximum amount of time each mouse is tested is 2 min (120 s), and if a mouse fails to remove the sticker it scores 120 s. The testing was performed over 5 trials with mice having a short break between trials and mean of times were considered for subsequent analyses. The base line was measured before starting the OligoGM1 administration and, throughout treatment mice were weekly tested.

*Pole climbing test* was used to test motor coordination and balance^9,13,38^. A wooden pole (diameter 8 mm, height 55 cm) was tilted at 45° angle with respect to the floor. The times spent by each mouse to ascend, turn around and descend the pole is measured. Mice were trained two times 2 hours before the test to allow familiarization with the equipment. Mice were tested three times and the times were averaged for each mouse. The pole climbing test was performed at day 27 of treatment, to prevent learning from compromising the success of the test.

### Neurochemical analysis

The levels of DA, DOPAC and norepinephrine were determined by measuring their tissue content in the striatum of animals after 4 weeks of treatment as previously reported^64,65^. Briefly, mice were anesthetized with IP injection of ketamine (100 mg/kg)/xylazine (10 mg/kg), and were decapitated. Striatal tissue was rapidly dissected on ice, weighed and frozen at −80 °C for future analysis. Upon thawing, each striatum sample (both side) was homogenized on ice with a mechanical stirrer (Mechanical Stirrer – Heavy duty, ISOLAB, Laborgerate GmbH, electronic speed settled at 300 rpm) for 30 sec in 0.1 M perchloric acid and 100 μM EDTA (20 μL/mg wet tissue). Homogenates were centrifuged at 14,000 × *g* for 20 min. Catecholamine content (sample vol. = 20 μL) of the resulting supernatants were quantified by HPLC–ED. The HPLC-ED system used was HTEC-500 (EiCOM USA, San Diego, CA, USA) that was equipped with a reverse-phase column (Varian, Brownlee, RP-18, Velosep, 3 μm, C18, 100 Å), and graphite working electrode (WE-3G, EiCOM USA, San Diego, CA, USA). Neurotransmitters were separated on a Velosep RP-18 column (100 × 3.2 mm; Applied Biosystems, Inc., Foster City, CA, USA) and quantified by measuring oxidative current at a glassy carbon wall-jet electrode (WE-GC model; Amuza Neuroscience) set at + 400 mV versus an Ag/AgCl electrode coupled to a potentiostat (RE-500; Amuza Neuroscience). The mobile phase consisted in 0.1 M sodium acetate buffer, pH 4.2, 0.1 mM EDTA, 1.2 mM sodium octyl sulfate, 8.0% (v/v) methanol. A solvent delivery pump (model HTEC-510; Amuza Neuroscience) delivered the mobile phase at 0.7 mL/min. Retention time was used to identify elements of interest, which were quantified on the basis of the peak height of oxidative current. The detection limit of the assay was 0.7 pg DA/sample.

### Fluorescent immunohistochemistry (IHC), imaging and analysis

Mice were deeply anesthetized with IP injection of ketamine (100 mg/kg)/xylazine (10 mg/kg), and transcardially perfused with 0.1 M PBS (23 °C, pH 7.4), followed by freshly prepared ice-cold 4% PFA in PBS. Whole brains were isolated, post-fixed overnight in 4% PFA at 4 °C and soaked in cryoprotective solution (30% sucrose in PBS) until tissue sinking. Brains were snap frozen in pre-cooled isopentane upon OCT embedding and stored at −80 °C, prior to cryostat sectioning. Tissues were sectioned (16 μm) using cryostat (MC 5050 Semi-automatic Cryostat, Histo-Line Laboratories) after OCT compound embedding in dry ice. Sections through the rostro-caudal extent of the *substantia nigra* were collected and every fifth section from *substantia nigra* was processed for TH and α-syn IHC analysis. SNpc was used for the IHC detection of TH and α-syn, as specifically showed in Fig. 7S of Supplementary Files. For immunofluorescence, 16 μm slices were treated for 1 h with citrate buffer and heat in a water bath at 80 °C. Then the slices were put in a blocking solution (1 h, 23 °C) containing 10% fetal calf serum and 0.25% Triton X-100 in PBS. After blocking, samples were incubated with the primary antibody diluted with the same solution overnight at 23 °C. The slices were then incubated with the secondary antibody (1 h, 23 °C), followed by nuclei staining with DAPI^66^ (1:5000, 15 min, 23 °C).

Primary antibodies for the following epitopes were used: anti-TH Rabbit pAb (1:500; Merck Millipore Cat# 657012-100UL, RRID:AB_10681344), and anti-α-syn Mouse IgG1 (1:1000; BD Biosciences Cat# 610787, RRID:AB_398108). The following secondary antibodies were used: goat anti-Rabbit IgG (H + L) antibody (1:500; Alexa Fluor 488, Thermo Fisher Scientific Cat# A-11008, RRID:AB_143165) and goat anti-Mouse IgG1 antibody (1:500; Alexa Fluor 568, Thermo Fisher Scientific Cat# A-21124, RRID:AB_2535766).

Confocal images were acquired using a Leica TCS SP5 laser scanning confocal microscope (Leica Microsystems, GmbH) using a HCX PL APO 40× (NA 1.25) Oil immersion objective. Tiff images were then imported and analyzed using ImageJ software (ImageJ, NIH http://rsb.info.nih.gov/ij/).

### Immunoblotting analysis

Freshly collected brains from PBS perfused mice were employed and the midbrain cut into 250 μm coronal sections with a vibratome sectioning system in cold PBS. The entire *substantia nigra* region, including pars compacta and reticulata, was isolated by dissecting under the microscope. The pooled sections were extracted with 1 mL/100 mg tissue of Cell Lysis Buffer (Cell Signaling, Danvers, MA). Aliquots containing 30 μg protein were denatured with Laemmli sample buffer (final concentration 0.1 M DTT, 63 mM Tris-HCl, 10% glycerol v/v, 2% SDS w/v, 0.01% blue bromophenol v/v), boiled at 100 °C for 5 min, separated on 4–20% polyacrylamide gels, and transferred to PVDF membranes using the Trans-Blot Turbo Transfer System (Bio-Rad). PVDF membranes were blocked with 5% milk (w/v) in TBS-0.1% tween (v/v) at 23 °C for 1 hour under gently shaking. TH and calnexin levels were assayed incubating the PVDF membranes respectively with primary anti-TH mouse antibody (1:2000 in 5% milk (w/v) in TBS-0.1% tween (v/v); Santa Cruz Biotechnology Cat# sc-25269, RRID:AB_628422), and anti-calnexin mouse antibody (1:1000 in 5% BSA (w/v) in TBS-0.1% tween (v/v), BD Biosciences Cat# 610524, RRID:AB_397884) overnight at 4 °C under gently shaking. Following, the reaction with secondary HRP-conjugated goat anti-mouse IgG (H + L) antibody (1:2000 in 5% milk (w/v) in TBS-0.1% tween (v/v), Thermo Fischer Scientific, Cat# RRID: AB_228307) at 23 °C for 1 in agitation and luminol detection (Cyanagen) were performed. Finally, the TH signal was normalized to calnexin signal. The data acquisition and analysis were performed using Alliance Uvitec (Cleaver Scientific Ltd, UK).

### Ganglioside analysis

Cortex and cerebellum were collected from WT and *B4galnt1*^+/−^ mice treated with BSS or OligoGM1 and subjected to lyophilization. The extraction of total lipids from lyophilized tissue was carried out with the solvent system chloroform/methanol/water in proportion of 20:10:1 by vol (1.5 mL/50 mg dry tissue) mixing samples at 23 °C in a thermomixer (Eppendorf) at 1400 rpm for 15 min. Total lipid extract was separated from the pellet by centrifugation at 13000 × *g* for 15 min, followed by a second and third extractions with chloroform/methanol, 2:1 by vol^39–41,67^. Total lipid extracts were subjected to a two phase partitioning by adding 20% water resulting in the separation of an aqueous phase containing gangliosides and in an organic phase containing all the other lipids after centrifugation at 13000 × *g* for 15 min at 23 °C^68^. Following a dialysis step to remove salts, gangliosides contained in the dialyzes aqueous phase, corresponding to 300 μg of tissue proteins, were resolved by mono-dimensional HPTLC using the solvent system chloroform/methanol/0.2% calcium chloride 50:42:11 by vol^69–72^. Following solvent evaporation, gangliosides were recognized by specific detection spraying with Ehrlich reagent, which is specific for sialic acid, and heating at 120 °C for 15 min. The relative amount of each ganglioside, taking into account its sialic acid content, was determined by densitometry using ImageJ software (NIH, Bethesda, USA; http://rsbweb.nih.gov/ij/).

### [^3^H]OligoGM1 penetration into the brain

To assesses the capability of OligoGM1 to reach the brain, five WT mice *(n* = 5, male) weighing 25 g each were IP injected with [^3^H]OligoGM1 (specific radioactivity: 2.2 Ci/mmol). A total of 6.5 × 10^7^ dpm of [^3^H]OligoGM1 was gently dried under nitrogen and dissolved in BSS containing 2.5 mg of cold OligoGM1. Each mouse was injected with 1.3 × 10^7^ dpm of [^3^H]OligoGM1 and 0.5 mg of cold OligoGM1 corresponding to 20 mg/Kg of OligoGM1. Following 24 h from the injection, animals were euthanized by heart perfusion with saline solution to remove the blood^73^. Immediately the brain, without cerebellum, was collected, weighted (±350 mg) and homogenized with 1.4 mL of cold distilled water (rescue 100 mg/400 mL) by 1 min treatment with a mechanical stirrer (Mechanical Stirrer – Heavy duty, Isolab, Laborgerate GmbH, electronic speed settled at 1200 rpm) at 4 °C (cold room).

#### Determination of radioactivity

Brain homogenates were submitted to determination of radioactivity by liquid scintillation counting. Specifically, 100 μL of brain homogenate were combined with 5 mL of ULTIMA GOLD liquid (PerkinElmer), shaken and counted for 20 minutes by Liquid Scintillation Analyzer (TRI-CARB 2100TR, Packard). For each brain homogenate three samples were counted (*n* = 3). Counting of tritium was carried with a program calculating the ratio of counts between two regions of energy spectrum to detect spectral shift and thus quenching. In the case of tritium, the regions are typically set from 0 to 18.6 keV in Region A (counting cpm A) and 2 to 18.6 in Region B (counting cpm B). Based on the efficiency of the instrument, usually around 70%, cpm A and cpm B are converted automatically (by a mathematic integration) into dpm. In our case the efficiency of the instrument is 63.09% (H3 eff 0–18.6 keV) and cmp were transformed to dpm according to the curve determined with unquenched standard (#6008500/1215–111) provided by PerkinElmer. The radioactivity background was automatically subtracted from each sample using as a blank the brain homogenate from an untreated animal.

The value of the radioactivity determined in such way corresponded to volatile and non-volatile radioactivity (total radioactivity)^73^. To establish the specific amount of volatile and non-volatile radioactivity 100 μL of brain homogenate (3 samples from each brain, *n* = 3) were dried under nitrogen flux and resuspended in the same initial volume of cold distilled water (100 μL) and counted for radioactivity content as above. The difference between the value obtained from non-dry homogenate (total radioactivity) and dry homogenate corresponded to non-volatile radioactivity (Fig. S1 of Supplementary).

We recall that the OligoGM1 is tritium labeled at position 6 of external galactose. Any possible galactosidase activity can result in external galactose removal leading to loss of radioactivity from the penta-saccharide. Radiolabeled galactose may enter in hepatocytes and may be transformed into glucose. This latter enters in the glycolysis pathway finally producing tritiated water. We cannot exclude that a very minor portion of released galactose enters in glycoconjugate biosynthesis, but the largest part of non-volatile radioactivity corresponds to the original oligosaccharide, while the largest part of volatile radioactivity corresponds to water.

#### Metabolic stability

To verify that the non-volatile radioactivity indeed correspond to OligoGM1, 600 μL of the homogenized mixture was centrifuged at 12000 × *g* for 10 min at 4 °C and the supernatant containing the soluble fraction was lyophilized. The precipitate was solubilized in 600 μl of distilled water and counted for radioactivity content liquid scintillation counting as above. The lyophilized material was suspended in 600 μL of methanol. 60 μL of the methanol solution (1/10 of the starting material) was separated by HPTLC using the solvent system chloroform/methanol/0.2% calcium chloride 30:50:13 (v/v/v). The [^3^H]OligoGM1 was visualized with Beta-Imager 2000 instrument (Biospace) using an acquisition time of 16 h and identified using pure [^3^H]OligoGM1 as standard.

### Protein determination

Protein concentration of samples was assessed using a DC protein assay kit according to manufacturer’s instructions, using bovine serum albumin as standard^74^.

### Statistical analysis

Data are expressed as mean ± SEM and were analyzed for significance by one-way or two-way ANOVA test followed by Bonferroni’s multiple comparisons post-hoc test. The analysis was performed with Prism software (GraphPad Software v8, Inc. La Jolla, CA, USA). In all cases statistical significance was set at p < 0.05. All data are available upon request.

### Other analytical methods

NMR spectra were recorded with a Bruker AVANCE-500 spectrometer at a sample temperature of 298 K. NMR spectra were recorded in CDCl3 or CD3OD and calibrated using the trimethylsilyl signal as internal reference.

Ganglioside and oligosaccharide mass spectrometry analyses were performed in positive ESI-MS. MS spectra were recorded on a Thermo Quest Finnigan LCQTM DECA ion trap mass spectrometer, equipped with a Finnigan ESI interface; data were processed by Finnigan Xcalibur software system (Thermo Fisher Scientific, Waltham, MA, USA).

All reactions were monitored by HPTLC on silica gel 60 plates.

## Supplementary information

Supplementary information

## Data Availability

The datasets generated and analyzed during the current study are available from the corresponding authors on reasonable request.
