# Supplementary material for: Parkinson’s disease recovery by GM1 oligosaccharide treatment in the B4galnt1+/− mouse model
Source: Sci Rep. 2019 Dec 18;9:19330. doi: 10.1038/s41598-019-55885-2 (PMC6920361; doi:10.1038/s41598-019-55885-2)
Supplement: Supplementary file 1 — Supplementary information [file 41598_2019_55885_MOESM1_ESM.pdf]

Supplementary information

Parkinson's disease recovery by GM1 oligosaccharide treatment in the *B4galnt1*$^{+/-}$ mouse model

Elena Chiricozzi[1*], Laura Mauri[1], Giulia Lunghi[1], Erika Di Biase[1], Maria Fazzari[1], Margherita Maggioni[1], Manuela Valsecchi[1], Simona Prioni[1], Nicoletta Loberto[1], Diego Yuri Pomè[1], Maria Grazia Ciampa[1], Pamela Fato[1], Gianluca Verlengia[2,3], Stefano Cattaneo[2], Robert Assini[4], Gusheng Wu[5], Samar Alselehdar[5], Robert W. Ledeen[5] and Sandro Sonnino[1*].

[1]Department of Medical Biotechnology and Transcriptional Medicine, University of Milano, Italy
[2]School of Medicine, University Vita-Salute San Raffaele, Milano, Italy
[3]Department of Medical Sciences and National Institute of Neuroscience, University of Ferrara, Italy
[4]Rutgers, The State University of New Jersey, Newark, New Jersey, USA
[5]Division of Neurochemistry, Department of Pharmacology, Physiology & Neuroscience, Rutgers New Jersey Medical School, Newark, NJ, USA

* Correspondence to:
Sandro Sonnino, Professor
Elena Chiricozzi, PhD
Via Fratelli Cervi 93, 20090 Segrate (MI), Italy
Phone: +39 0250330360
Fax: +39 0250330365
sandro.sonnino@unimi.it
elena.chiricozzi@unimi.it

Running title:
Parkinson' recovery by GM1 oligosaccharide

ORCID ID
Elena Chiricozzi 0000-0001-7431-920
Sandro Sonnino 0000-0001-8180-5908

Methods

Immunoblotting analysis

Freshly collected brains from PBS perfused mice were employed and the midbrain cut into 250 μm coronal sections with a vibratome sectioning system in cold PBS. The entire *substantia nigra* region, including compact and reticulata, was isolated with a dissecting microscope. The pooled sections were extracted with 1 mL/100 mg tissue of Cell Lysis Buffer (Cell Signaling, Danvers, MA). Aliquots containing 30 μg protein were denatured with Laemmli sample buffer (final concentration 0.1 M DTT, 63 mM Tris-HCl, 10 % glycerol v/v, 2% SDS w/v, 0.01% blue bromophenol v/v), boiled at 100°C for 5 min, separated on 4–20% polyacrylamide gels, and transferred to PVDF membranes using the Trans-Blot® Turbo™ Transfer System (Bio-Rad). PVDF membranes were blocked with 5% milk (w/v) in TBS-0.1% tween (v/v) at 23 °C for 1 hour under gently shaking. Phosphorylated α-syn (S129) level and calnexin levels were assayed using respectively anti-phospho-α-syn (S129) rabbit antibody (1:1000 in 5% milk (w/v) in TBS-0.1% tween (v/v); Abcam Cat# ab51253, RRID:AB_869973) and anti-calnexin mouse antibody (1:1000 in 5% BSA (w/v) in TBS-0.1% tween (v/v), BD Biosciences Cat# 610524, RRID:AB_397884) followed by reaction with secondary HRP-conjugated antibodies anti-rabbit IgG antibody (1:2000, Cell Signaling Technology Cat# 7074, RRID:AB_2099233) and goat anti-mouse IgG (H+L) antibody (1:2000, Thermo Fischer Scientific, Cat# RRID: AB_228307) and luminol detection. Finally, the phospho-α-syn (S129) signal was normalized to calnexin signal. The data acquisition and analysis were performed using Alliance Uvitec (Cleaver Scientific, Ltd, UK).

Fluorescent immunohistochemistry (IHC), imaging and analysis

Mice were deeply anesthetized with with IP injection of ketamine (100 mg/kg)/xylazine (10 mg/kg), perfused with 0.1 M PBS (23°C, pH 7.4), followed by freshly prepared ice-cold 4% PFA in PBS. Whole brains were isolated, post-fixed overnight in 4% PFA at 4°C and soaked in cryoprotective solution (30% sucrose in PBS) until tissue sinking. Brains were snap frozen in pre-cooled isopentane upon OCT embedding and stored at -80°C, prior to cryostat sectioning. Tissues were sectioned (16 μm) using cryostat (MC 5050 Semi-automatic Cryostat, Histo-Line Laboratories) compound embedding in dry ice. Sections through the rostro-caudal extent of the *substantia nigra* were collected and every fifth section from *substantia nigra* was processed for phosphorylated α-syn (S129) IHC analysis. SNpc was used for the IHC detection of phosphorylated α-syn (S129), as specifically indicated in Fig. S7 of supplementary files. For immunofluorescence, 16 μm slices were treated for 1 h with citrate buffer and heat in a water bath at 80°C. Then the slices were put in a blocking solution (1h, 23°C) containing 10% fetal calf serum and 0.25% Triton X-100 in PBS. After blocking, samples were incubated with the primary antibody diluted with a same solution overnight at 23°C. The slices were then incubated with the secondary antibody (1h, 23°C), followed by nuclei staining with DAPI[1] (1:5000, 15 min, 23°C).

Anti-phospho-α-syn (S129) rabbit antibody (1:100; Abcam Cat# ab51253, RRID:AB_869973) and goat anti-Rabbit IgG (H+L) antibody (1:500; Alexa Fluor 488, Thermo Fisher Scientific Cat# A-11008, RRID:AB_143165) were used as primary and secondary antibodies respectively.

Immunofluorescence images were acquired using a NikonEclipse Ni upright microscope (400x magnification) and a Nikon DIGITAL SIGHT DS-U1 CCD camera. Tiff images were then imported and analyzed using ImageJ software (ImageJ, NIH http://rsb.info.nih.gov/ij/).

Supplementary Figure 1

WT mice (*n*=5)	[^{3}H]OligoGM1 administered	Brain radioactivity (total radioactivity)	*Volatile radioactivity*	*Non-volatile radioactivity*
I	$1.3x10^7$ dpm	$3.38x10^6$	$5.07x10^5$	$2.87x10^6$
II	$1.3x10^7$ dpm	$3.51x10^6$	$5.96x10^5$	$2.91x10^6$
III	$1.3x10^7$ dpm	$3.9x10^6$	$6{,}24x10^5$	$3{,}27x10^6$
IV	$1.3x10^7$ dpm	$2.6x10^6$	$4{,}94x10^5$	$2{,}1x10^6$
V	$1.3x10^7$ dpm	$2.86x10^6$	$4{,}86x10^5$	$2{,}37x10^6$
average		$3.25x10^6$ (± 25% of [^{3}H]OligoGM1administered)	$5.41x10^5$ (± 16% of total radioactivity)	$2{,}7x10^6$ (± 84% of total radioactivity)

Supplementary Fig. 1 Radioactivity distribution into the brain. Five WT mice (*n*=5, male, 25 g each) were singly IP injected with $1.3x10^7$ dpm of [^{3}H]OligoGM1 plus 0.5 mg of cold OligoGM1 corresponding to 20mg/Kg of OligoGM1. After 24 h following injection, animals were euthanized by heart perfusion with saline solution to remove the blood[2]. Immediately the brain, without cerebellum, was collected, weighted (± 350 mg) and homogenized as reported in Methods section.

Brain homogenate was submitted to determination of total radioactivity by liquid scintillation corresponding to volatile and non-volatile radioactivity (total radioactivity)[2]. For each brain homogenate tree replicates were counted (*n*=3). To establish the specific amount of volatile and non-volatile radioactivity, the brain homogenate (3 samples from each brain, *n*=3) was dried under flux of nitrogen, resuspended in cold distilled water and counted for radioactivity content as above. The difference between the value obtained from not-dry homogenate (total radioactivity) and dry homogenate corresponded to non-volatile radioactivity. The data reported (total radioactivity and volatile radioactivity) are the mean value of three counts for each brain homogenate dried or not.

Supplementary Figure 2

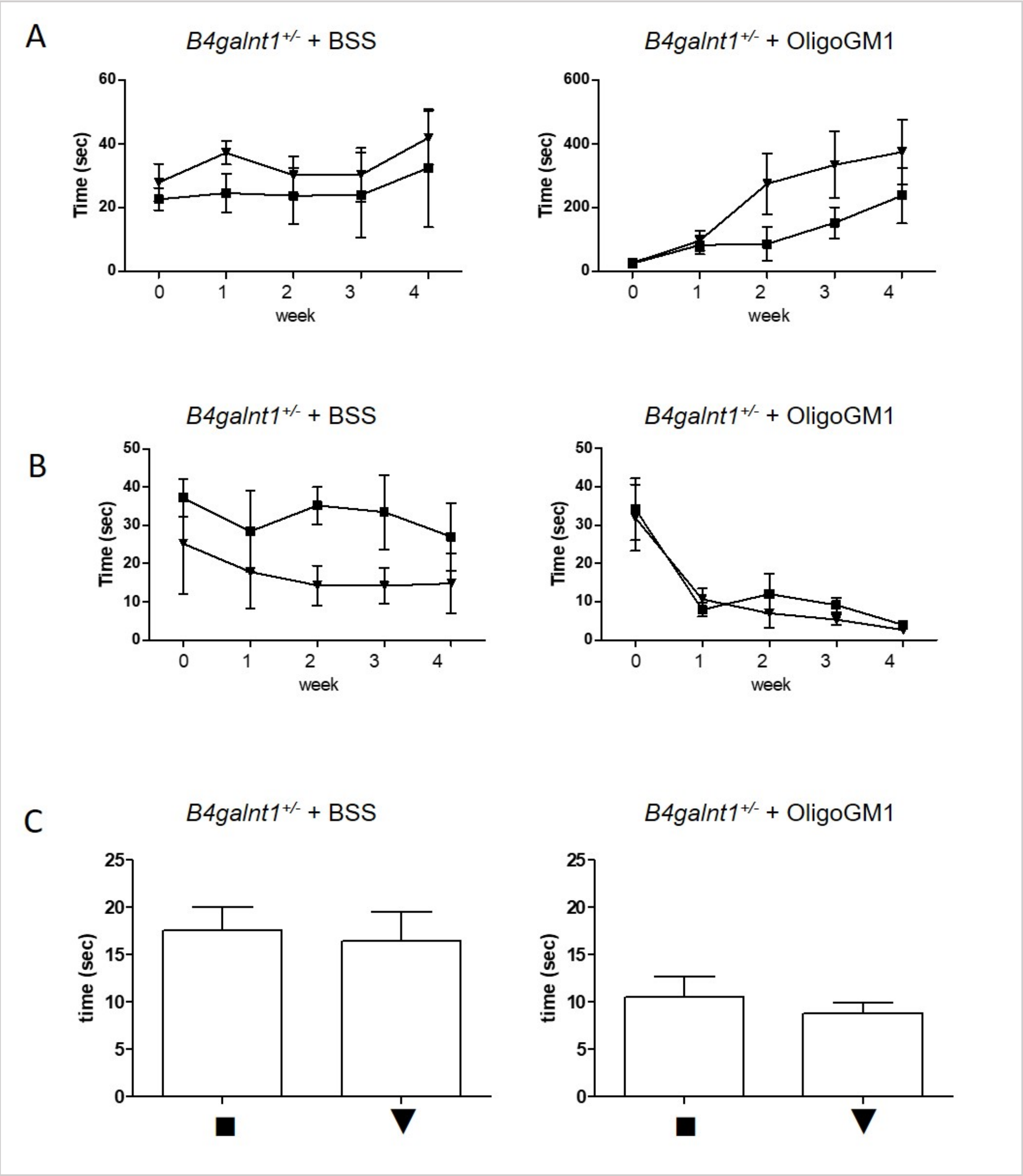

Supplementary Fig. 2 Gender effect on behavioral response upon treatment. Data are expressed as mean ± SEM [male (*n*=6), female (*n*=6); two-way ANOVA followed by Bonferroni's multiple comparisons test] **a)** Grip duration Test. Left: *B4galnt1*$^{+/-}$ mice + BSS no significant difference between ■ male and ▼ female; Right *B4galnt1*$^{+/-}$ mice + GM1 oligosaccharide no significant difference between ■ male and ▼ female; **b)** Irritant Removal Test. Left: *B4galnt1*$^{+/-}$ mice + BSS no significant difference between ■ male and ▼ female; Right *B4galnt1*$^{+/-}$ mice + GM1 oligosaccharide no significant difference between ■ male and ▼ female; **c)** Pole Climbing Test. Left: *B4galnt1*$^{+/-}$ mice + BSS no significant difference between ■ male and ▼ female; Right *B4galnt1*$^{+/-}$ mice + GM1 oligosaccharide no significant difference between ■ male and ▼ female.

Supplementary Figure 3

A

	WT + BSS	*B4galnt1*$^{+/-}$ + BSS	*B4galnt1*$^{+/-}$ + OligoGM1
GM1	23,4	22,4	22,1
GD3	1,9	5,1	5,6
GD1a	38,0	27,9	27,6
GD1b	9,8	14,7	15,4
GT1b	22,8	25,8	25,2
GQ1b	4,1	4,1	4,1

B

	WT + BSS	*B4galnt1*$^{+/-}$ + BSS	*B4galnt1*$^{+/-}$ + OligoGM1
GM1	21,7	19,0	21,0
GD3	3,4	13,9	14,6
GD1a	18,7	9,9	8,4
GD1b	12,0	12,2	13,3
GT1b	34,8	36,2	34,2
GQ1b	9,4	8,8	8,5

Supplementary Fig. 3 Ganglioside distribution in **a)** cortex and **b)** cerebellum for WT + BSS (*n*= 5), *B4galnt1*$^{+/-}$ + BSS (*n*=8), and *B4galnt1*$^{+/-}$ + OligoGM1 (*n*=8). Data are expressed as percentage distribution of ganglioside/mg protein.

Supplementary Figure 4

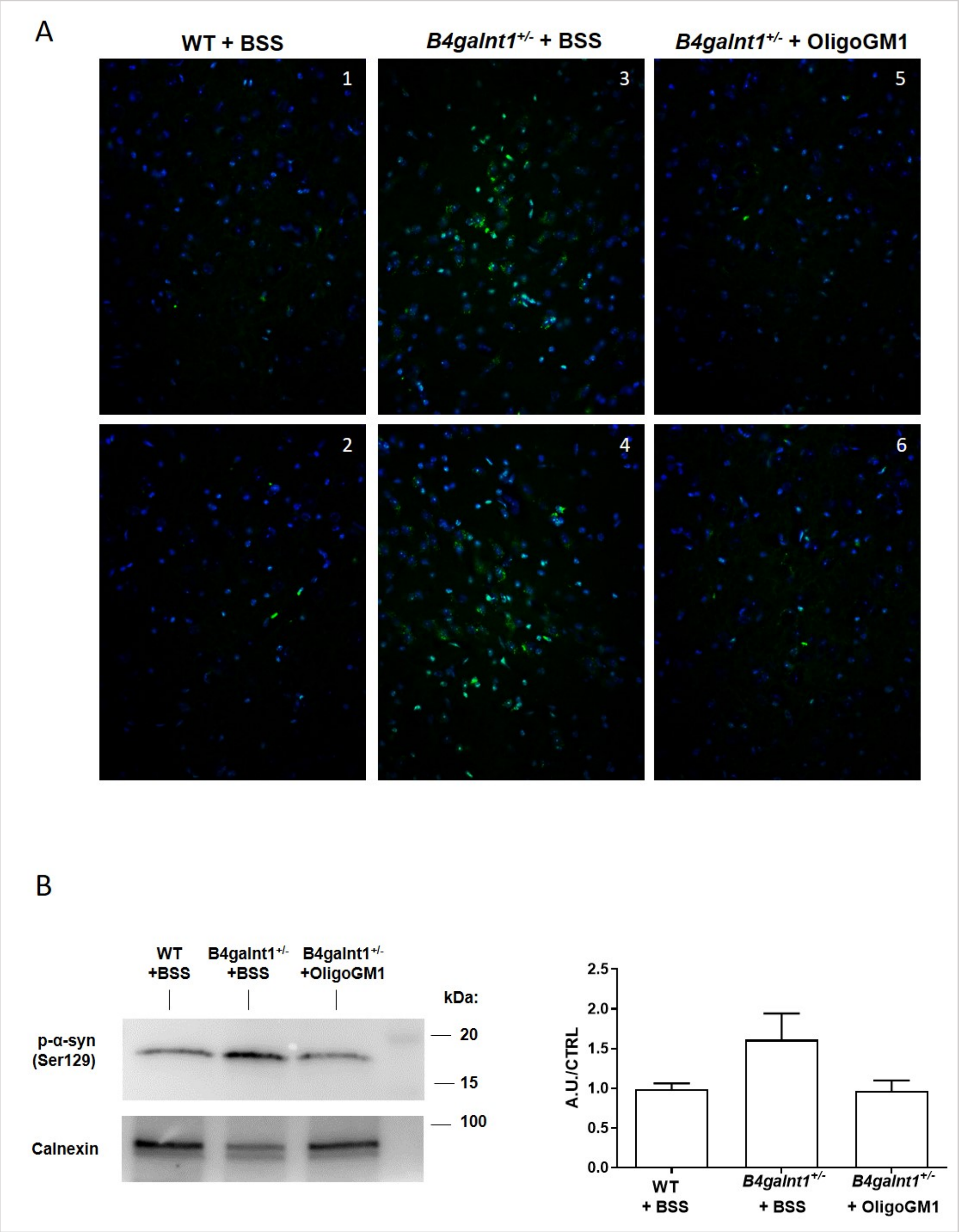

Supplementary Fig. 4 Phosphorylated α-syn (S129) detection **a)** Fluorescent IHC analysis. Representative IHC images of S129 phosphorylated α-syn (green) and nuclei (blue) immunolabeling in the SNpc. 1-2) WT + BSS (*n*=3); 3-4) *B4galnt1*$^{+/-}$ + BSS, (*n*=3) 5-6) *B4galnt1*$^{+/-}$ + OligoGM1, *n*=3). **b)** Immunoblotting analysis. On the left: representative immunoblotting images of S129 phosphorylated α-syn (p-α-syn) and calnexin are shown after cropping (full length images of blot are presented as supplementary Fig. S8). On the right: Semi-quantitative analysis of nigral p-α-syn (S129) expression related to calnexin level (*n*=3). Data are expressed as fold increase over the control (WT) of the mean ± SEM.

Supplementary Figure 5

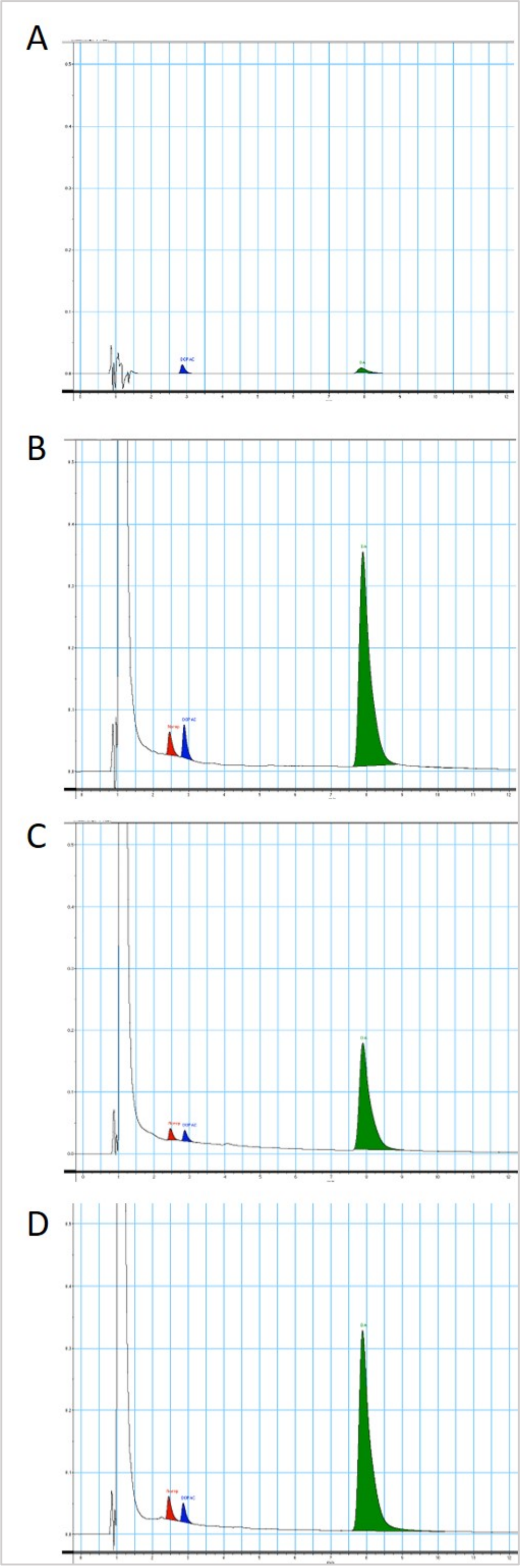

Supplementary Fig. 5 ED-HPLC Chromatogram picks representative images [red peak: norepinephrine; blue peak: DOPAC and green peak: DA] **a)** Standard; **b)** WT + BSS; **c)** *B4galnt1*$^{+/-}$ mice + BSS; **d)** *B4galnt1*$^{+/-}$ mice + OligoGM1.

Supplementary Figure 6

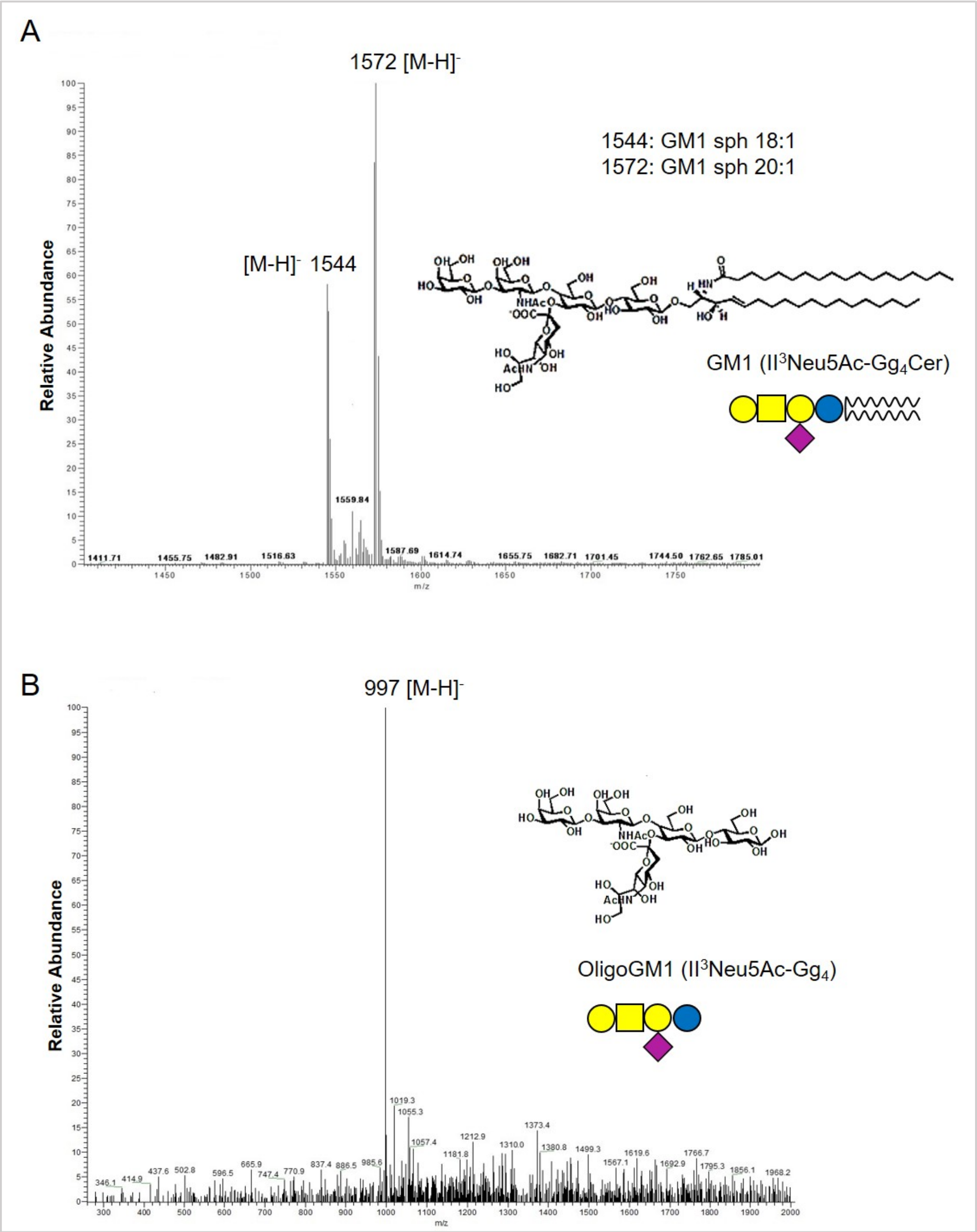

Supplementary Fig. 6 GM1 was purified from a mixture of bovine extract gangliosides using the ion-exchange chromatography technique[3]. Subsequently, GM1 underwent catalytic ozonolysis and base treatment with triethylamine to cleave the apolar chain from the oligosaccharide core. OligoGM1 was purified from the reaction mixture using standard chromatographic procedures and its purity was proved by MS analysis. Ganglioside nomenclature is in accordance with IUPAC-IUBB recommendations[4]. Glycoconjugates representation is according to Varky *et al.*[5]. **a)** Left: MS profile of GM1 ganglioside: ESI-MS (negative-ion mode): m/z= 1544 and 1572 $[M - H]^-$; Right: GM1 structure. **b)** Left: MS profile of GM1 oligosaccharide: ESI-MS (negative-ion mode): m/z= 997 $[M - H]^-$; Right: OligoGM1 structure.

Supplementary Figure 7

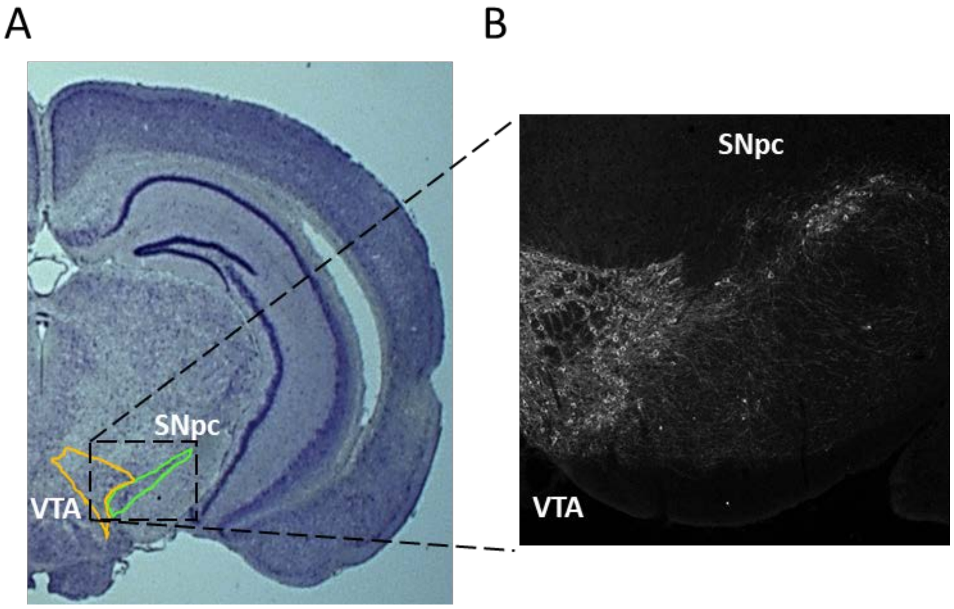

Supplementary Fig. 7 Illustration indicating the SNpc used for IHC analysis. **a)** Representative image of coronal section of mouse brain stained for Nissl from our own images archive (images were acquire using a NikonEclipse Ni upright microscope, 2.5x magnification); **b)** Representative immunofluorescence image of SNpc section from *B4galnt1*$^{+/-}$ + OligoGM1 mouse brain immunostained for TH (images were acquired using a Zeiss Axio Observer.Z1 with Hamamatsu EMCCD 9100 – 02, using a EC Plan - APOCHROMAT 5X (NA 0.16) Dry).

Supplementary Figure 8

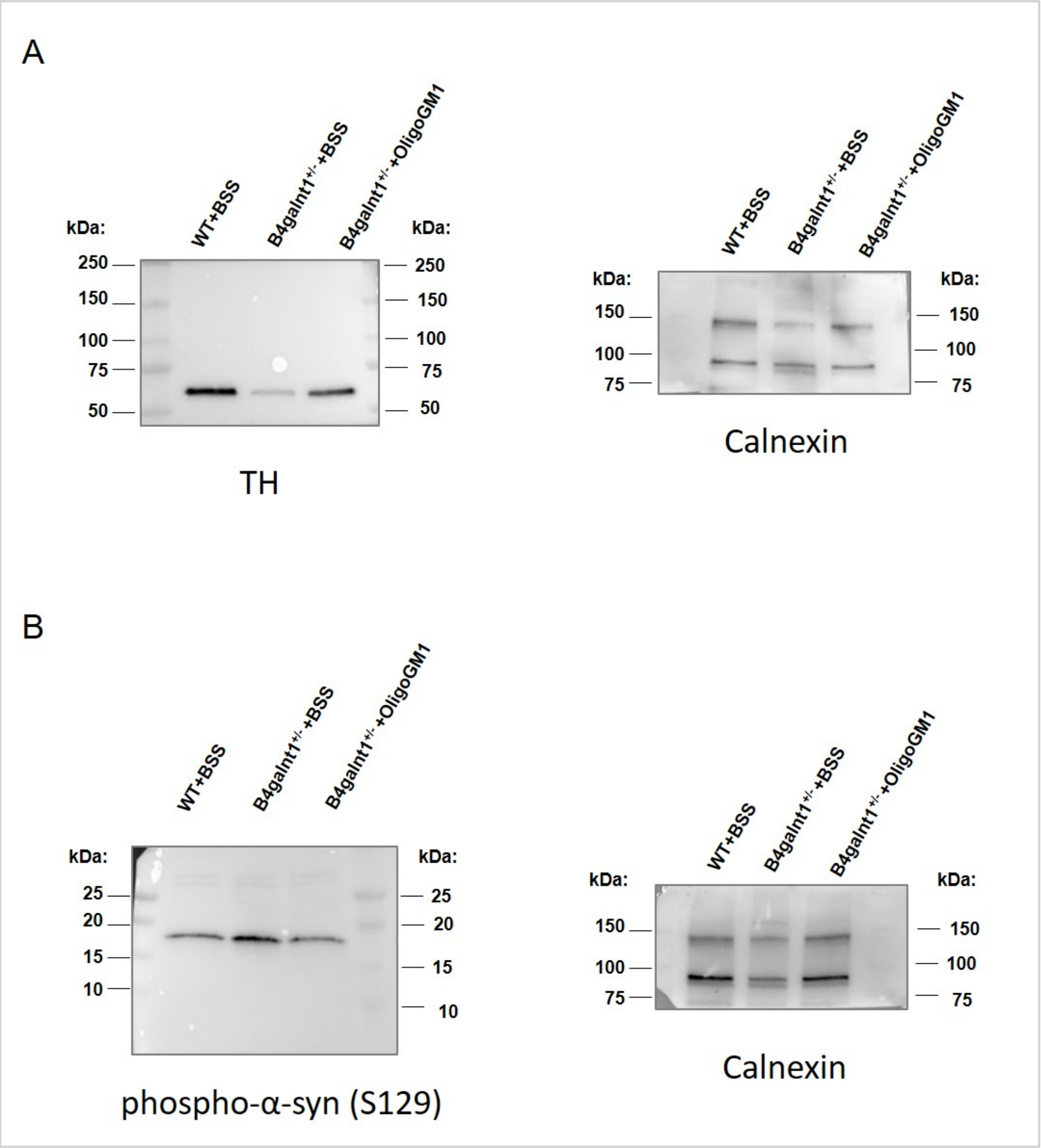

Supplementary Fig. 8 Full length western blots of images presented in Figure 7 **(a)** and in Supplementary Figure 4 **(b)**.

References

1. Chentanez, V. et al. Ganglioside GM1 (porcine) ameliorates paclitaxel-induced neuropathy in rats. J. Med. Assoc. Thai. 92, 50-7 (2009)
2. Orlando, P. et al. The fate of tritium labeled GM1 ganglioside injected in mice. Pharmacol. Res. Com. 11, 759-773 (1979).
3. Acquotti, D., Cantu, L., Ragg, E. & Sonnino, S. Geometrical and conformational properties of ganglioside GalNAc-GD1a, IV4GalNAcIV3Neu5AcII3Neu5AcGgOse4Cer. Eur. J. Biochem. 225, 271-288 (1994).
4. IUPAC-IUMB JCoBN. Nomenclature of glycolipids. Carbohydr. Res. 312, 167-175 (1998).
5. Varki, A. et al. Symbol nomenclature for graphical representations of glycans. Glycobiology 25, 1323-1324 (2015).
